# Efficient Bayesian inference under the multispecies coalescent with migration

**DOI:** 10.1073/pnas.2310708120

**Published:** 2023-10-23

**Authors:** Tomáš Flouri, Xiyun Jiao, Jun Huang, Bruce Rannala, Ziheng Yang

**Affiliations:** ^a^Department of Genetics, Evolution, and Environment, University College London, London WC1E 6BT, United Kingdom; ^b^Department of Statistics and Data Science, China Southern University of Science and Technology, Shenzhen 518055, China; ^c^Department of Intelligent Medical Engineering, School of Biomedical Engineering, Capital Medical University, Beijing 100069, China; ^d^Department of Evolution and Ecology, University of California, Davis, CA 95616

**Keywords:** BPP, gene flow, genomics, migration, multispecies coalescent

## Abstract

Analyses of genome sequence data have revealed pervasive interspecific gene flow and enriched our understanding of the role of gene flow in speciation and adaptation. Inference of gene flow using genomic data requires powerful statistical methods. Yet current likelihood-based methods involve heavy computation and are feasible for small datasets only. Here, we implement the multispecies-coalescent-with-migration model in the Bayesian program bpp, which can be used to test for gene flow and estimate migration rates, as well as species divergence times and population sizes. We develop Markov chain Monte Carlo algorithms for efficient sampling from the posterior, enabling the analysis of genome-scale datasets with thousands of loci. Implementation of both introgression and migration models in the same program allows us to test whether gene flow occurred continuously over time or in pulses. Analyses of genomic data from *Anopheles* mosquitoes demonstrate rich information in typical genomic datasets about the mode and rate of gene flow.

## Significance

Inference of gene flow using genomic data requires powerful methods as the process of coalescent, migration, and mutation is highly stochastic. However, it is challenging to implement the multispecies coalescent with migration (MSC-M) model in a full likelihood framework correctly and efficiently. We developed Markov chain Monte Carlo algorithms under the MSC-M model and implement them in our Bayesian program bpp to achieve efficient computation. We conduct extensive validations and tests and show that our implementation is reliable and can handle large datasets with thousands of loci. We analyzed genomic data from the *Anopheles* mosquitoes to demonstrate the feasibility of using genomic data to test for gene flow and to estimate the rate of gene flow.

One of the most important findings arising from comparative analyses of sequenced genomes during the past two decades is the prevalence of interspecific gene flow. Hybridization has been inferred in both plants [e.g., *Arabidopsis* (1)] and animals, including *Anopheles* mosquitoes (2), *Panthera* cats (3), cichlid fishes (4), and Hominins (5). Hybridization transfers genetic variation across species and by creating new allelic combinations at multiple loci may contribute to ecological adaptation (6, 7). Inference of gene flow can further our understanding of speciation (8, 9), help delineate species boundaries (10, 11), and guide efforts to conserve biodiversity and detect invasive species.

Gene flow is often inferred using simple summaries of genomic data. For example, the D statistic (12) and HyDe (13) test for gene flow using genome-wide site-pattern counts in a species quartet, while SNaQ (14, 15) uses the reconstructed gene tree topologies. These methods have low power and are often unable to identify gene flow between sister lineages, or to infer the direction, timing, and strength of gene flow (16–18).

Likelihood methods based on the multispecies coalescent (MSC) model (19) make full use of information in the data, providing rich inference using genomic datasets (20). Two simple models of gene flow have been developed under the MSC, representing different modes of gene flow (16, 17). The MSC-with-introgression [MSC-I; (21)] model, also known as multispecies network coalescent [MSNC, (22, 23)], assumes that gene flow occurs at a particular time point in the past. The MSC-with-migration (MSC-M) model, also known as the isolation-with-migration (IM) model, assumes that gene flow occurs continuously at a certain rate every generation after species divergence (24, 25). In both models the rate of gene flow should be considered an ‘effective’ rate, reflecting the combined effects of gene flow and natural selection on introgressed alleles, influenced by genetic drift and local recombination rate (9). The two models are simple extremes as in reality the rate of gene flow may be expected to vary over time (26). Here, we focus on MSC-M.

Under the MSC-M model, the gene genealogy at any locus includes not only the tree topology and coalescent times (branch lengths) but also detailed migration history (the number, directions, and timings of migration events). There may be no upper limit to the number of migration events at each gene locus. Likelihood inference has to average over the gene genealogy underlying the sequence data at each locus, including the migration history. There have been two approaches to dealing with the migration history (16). The first relies on a theory developed in the structured coalescent framework, in which the backward-in-time process of coalescence and migration is described using a continuous-time Markov chain, to integrate out the migration history at each locus analytically (27–30). However, the number of states in the Markov chain grows explosively with the increase in the number of species and the number of sequences (30). Thus, this approach is feasible for very small numbers of species and sequences but can deal with many loci. The maximum likelihood program 3s (29, 31) is limited to three species and three sequences per locus. The program mist (32) is implemented for two species/populations only and can handle eight sequences per locus. Both programs can handle >10,000 loci.

The second approach uses Markov chain Monte Carlo (MCMC) to average over the gene trees including the migration history numerically. Both IMa3 (24, 25) and G-PhoCS (33) take this strategy. While G-PhoCS assumes that the MSC-M model is fixed, IMa3 also allows the species phylogeny to change during the MCMC. Both programs involve a high computational load: IMa3 has been used to analyze data of a few hundred loci while G-PhoCS has been used to analyze data of a few thousand.

Population genetic models of population subdivision and migration (34) have been implemented in the program migrate (35–37). A major difference of these models from MSC-M is that they do not account for the population/species phylogeny or the history of population divergences. They can be considered special cases of MSC-M with divergence times approaching ∞ (see below). The structured coalescent model was also implemented in the program beast2 as the MultiTypeTree package (38), and approximations were made to improve computation in the mascot package (39). These are designed for phylodynamic analysis of viral sequence data, treating geographical locations as subpopulations, with the aim of estimating migration rates, reconstructing transmission histories, and tracing the emergence of outbreaks in a pandemic (38). The so-called “mugration” model treats migrations between geographical regions as a continuous-time Markov chain, such as used to model mutations, and assumes that the migration process does not influence the shape of the genealogical tree. The model thus has major deviations from the structured coalescent or MSC-M, leading to unreliable inference of migration rates and high sensitivity of the inferred root location to sampling biases (40). They do not appear to be suitable for analysis of multilocus sequence alignments from different species under the MSC.

Overall, current likelihood methods under the MSC-M model for multilocus sequence data including both IMa3 and G-PhoCS involve heavy computation and do not scale well with genomic datasets. The algorithmic challenge is not mainly due to the expanded state space because of migration histories at multiple loci; rather, it lies in the extremely stringent constraints placed by the gene trees on the species tree or on the MSC-M model. For example, if we propose to modify a species divergence time with all gene trees fixed, only tiny changes are permissible. In the context of MSC without gene flow, we have found that smart MCMC moves that make coordinated changes to both the species tree and the gene trees can dramatically improve MCMC mixing (19, 41, 42), making it possible to analyze datasets with >10,000 loci (21, 43).

Here, we implement the MSC-M model in bpp, a coalescent-based Bayesian MCMC program (44, 45). We develop MCMC algorithms for efficient mixing and conduct extensive simulations to validate our algorithms. We also show that the MSC-M model can be used to analyze classical population genetic models of subdivision and structure such as the finite-island model (46, 47) and the stepping-stone model (48) which are special cases of the MSC-M model. We show that bpp outperforms existing methods in both reliability and scalability. We applied both MSC-M and MSC-I models to genomic data from the *Anopheles gambiae* group of African mosquitoes (2, 43), to test for gene flow and to estimate the rate of gene flow, as well as other major population parameters such as species divergence times and population sizes. Having both MSC-I and MSC-M in the same program allows us to examine their differences when both are applied to the same data and to compare their goodness of fit.

## Results

### Gene-tree Density under the MSC-M Model.

**Fig. 1.**
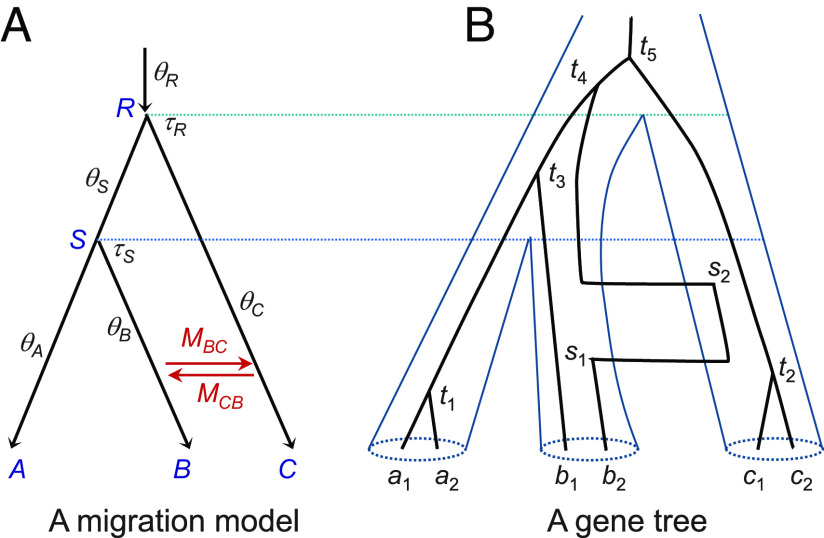
(*A*) A species tree for three species (A,B,C) with migration between species B and C showing model parameters, Θ = ($\tau_{R}$, $\tau_{S}$, $\theta_{A}$, $\theta_{B}$, $\theta_{C}$, $\theta_{R}$, $\theta_{S}$, $M_{\mathrm{BC}}$, $M_{\mathrm{CB}}$). There are three sets of parameters in the model: species divergence times ($\tau_{R}\equiv \tau_{\mathrm{ABC}}$, $\tau_{S}\equiv \tau_{\mathrm{AB}}$), population sizes ($\theta_{A}$, $\theta_{B}$, $\theta_{C}$, $\theta_{R}$, $\theta_{S}$), and migration rates ($M_{\mathrm{BC}},M_{\mathrm{CB}}$). Both τ and θ are measured in the expected number of mutations per site. The (population) migration rate is defined as $M_{\mathrm{ij}}=N_{j}m_{\mathrm{ij}}$, the expected number of migrants from species i to j per generation, where $N_{j}$ is the (effective) population size of species j and $m_{\mathrm{ij}}$ is the proportion of immigrants in population j from population i. (*B*) A possible gene tree with the complete history of coalescent and migration events at a locus with two sequences from each of the three species ($a_{1},a_{2}$ from A; $b_{1},b_{2}$ from B; and $c_{1},c_{2}$ from C). In the backward-in-time process of coalescent and migration, the five coalescent events occur at times $t_{1}$–$t_{5}$, while sequence $b_{2}$ experienced two migration events from B to C at time $s_{1}$ and back from C to B at time $s_{2}$, with $t_{1}<t_{2}<s_{1}<s_{2}<t_{3}<t_{4}<t_{5}$.

Fig. 1*A* illustrates the MSC-M model, which involves three sets of parameters: species divergence times (τ), population sizes (θ), and migration rates (M), with Θ={τ,θ,M}. Time is scaled by mutations so that one time unit is the expected time to accumulate one mutation per site. Thus, both τ and θ are measured in expected number of mutations per site. At this time scale, two sequences from a population of size θ coalesce at the rate of $\frac{2}{\theta }$. Migration rate $M_{\mathrm{ij}}$ is defined as the expected number of migrants from species i to j per generation, with $M_{\mathrm{ij}}=N_{j}m_{\mathrm{ij}}$, where $N_{j}$ is the (effective) population size of species j and $m_{\mathrm{ij}}$ is the proportion of immigrants in population j from population i. Note that we use the real-world view (with time running forward) to define the migration rate parameter. Let $X=\{X^{\left(i\right)}\}$ denote the data, with $X^{\left(i\right)}$ to be the sequence alignment at locus i. Let $G=\{G^{\left(i\right)}\}$ be the gene trees, where $G^{\left(i\right)}$ includes the rooted tree, the coalescent times, and the migration history at the locus (including the number, directions, and timings of migration events). We assume no recombination among sites in the sequence of the same locus and free recombination between loci, so that all sites at the same locus share the same gene tree while gene trees at multiple loci are independent. A recent simulation suggests that inference under the MSC is robust to moderate levels of recombination (49).

We implement MCMC algorithms to sample from the joint conditional distribution of the parameters and the gene trees.

[1] $$\begin{array}{c} p(\Theta ,G|X)\propto p(\Theta )p(G|\Theta )p(X|G), \end{array}$$

where p(Θ) is the joint prior, p(G|Θ) is the probability density of the gene trees given the parameters in the MSC-M model, and p(X|G) is the probability of the sequence data given the gene trees or the phylogenetic likelihood (50).

The probability density of the gene trees under the MSC-M model, p(G|Θ) in Eq. **1**, is essentially given by the structured coalescent process of coalescent and migration (24, 27, 28, 34, 35) operating within each time interval between species divergences. To accommodate the species phylogeny, we simply reset the process when we reach a species divergence event, with an update to the number of populations, migration rates, and other population parameters. The MSC-M process is also a variable-rate (piecewise constant-rate) Poisson process, in which the coalescent and migration rates change at any coalescent event, migration event, and species divergence event (51).

We break the time period for each species j into $K_{\mathrm{ij}}$ time segments at locus i, during which the coalescent and migration rates are constant. Let $t_{\mathrm{ijk}}$ be the duration of the kth time segment, and $n_{\mathrm{ijk}}$ be the number of lineages, with $k=1,\cdots ,K_{\mathrm{ij}}$. Let $w_{\mathrm{sji}}$ be the number of migration events from species s to j at locus i (with time running forward). We define the indicator $I_{\mathrm{sjk}}$ to be 1 if migration from s to j is possible during time segment k (i.e., if both species s and j exist in time segment k and are permitted to exchange migrants) and 0 otherwise. The probability density for the gene trees under MSC-M is then a product over species and over loci, with the contribution from species j and locus i given by the variable-rate Poisson process, equal to the Poisson rates for coalescent and migration events that have occurred times the probability of no events occurring during the total time duration. Let $G_{j}$ be parts of the gene trees in species j (over all loci), and let $G=\{G_{j}\}$. Then,

[2] $$\begin{array}{cc} p(G|\Theta )= & \prod_{j}p\left(G_{j}|\Theta \right)=\prod_{j}\prod_{i}[(\frac{2}{\theta_{j}h_{i}}{)}^{c_{\mathrm{ij}}}\prod_{s}(\frac{4M_{\mathrm{sj}}}{\theta_{j}h_{i}}{)}^{w_{\mathrm{sji}}}\\ & \times \exp\{-\sum_{k=1}^{K_{\mathrm{ij}}}(\frac{n_{\mathrm{ijk}}\left(n_{\mathrm{ijk}}-1\right)}{\theta_{j}h_{i}}\\ & +\frac{n_{\mathrm{ijk}}\cdot 4\sum_{s}I_{\mathrm{sjk}}M_{\mathrm{sj}}}{\theta_{j}h_{i}})t_{\mathrm{ijk}}\}], \end{array}$$

where $h_{i}$ is the heredity/ploidy scalar for the ith locus (e.g., 1 for autosomes, $\frac{3}{4}$ for X-linked, $\frac{1}{4}$ for Y-linked or mtDNA loci), and $c_{\mathrm{ij}}$ is the number of coalescent events in species j at locus i. Here $\frac{2}{\theta_{j}h_{i}}$ is the coalescent rate per time unit for a pair of sequences in species j at locus i and $\frac{4M_{\mathrm{sj}}}{\theta_{j}h_{i}}$ is the (mutation-scaled) migration rate from species s to j, with time running forward.

For example, the density for the gene tree of Fig. 1*B* for a locus with six sequences is

[3] $$\begin{array}{cc} p(G|\Theta )= & \;[\frac{2}{\theta_{A}}{\;e\;}^{-\frac{2}{\theta_{A}}t_{1}}]\times [{\;e\;}^{-\frac{2}{\theta_{B}}\left(s_{1}+\tau_{S}-s_{2}\right)}\\ & \;\;\cdot \frac{4M_{\mathrm{CB}}}{\theta_{B}}{\;e\;}^{-\frac{4M_{\mathrm{CB}}}{\theta_{B}}\left[2\left(s_{1}+\tau_{S}-s_{2}\right)+\left(s_{2}-s_{1}\right)\right]}]\\ & \times [\frac{2}{\theta_{C}}{\;e\;}^{-\frac{2}{\theta_{C}}\left(t_{2}+s_{2}-s_{1}\right)}\\ & \cdot \frac{4M_{\mathrm{BC}}}{\theta_{C}}{\;e\;}^{-\frac{4M_{\mathrm{BC}}}{\theta_{C}}\left[2\left(t_{2}+s_{2}-s_{1}\right)+\left(s_{1}-t_{2}\right)+\left(\tau_{S}-s_{2}\right)\right]}]\\ & \times [\frac{2}{\theta_{S}}{\;e\;}^{-\frac{6}{\theta_{S}}\left(t_{3}-\tau_{S}\right)-\frac{2}{\theta_{S}}\left(\tau_{R}-t_{3}\right)}]\\ & \times [\frac{2}{\theta_{R}}\cdot \frac{2}{\theta_{R}}{\;e\;}^{-\frac{6}{\theta_{R}}\left(t_{4}-\tau_{R}\right)-\frac{2}{\theta_{R}}\left(t_{5}-t_{4}\right)}]. \end{array}$$

The five terms in the square brackets correspond to contributions from the five populations: A,B,C,S, and R, respectively. For species A, the contribution is $\frac{2}{\theta_{A}}{\;e\;}^{-\frac{2}{\theta_{A}}t_{1}}$, as there is one coalescent event, between $a_{1}$ and $a_{2}$ at time $t_{1}$. In species B, there is no coalescent, so the probability of having no coalescent when there were two sequences during the time periods ($0,s_{1}$) and ($s_{2},\tau_{S}$) is ${\;e\;}^{-\frac{2}{\theta_{B}}\left(s_{1}+\tau_{S}-s_{2}\right)}$. There is a migration event; hence, the rate $\frac{4M_{\mathrm{CB}}}{\theta_{B}}$, while the probability that no migration occurs during time periods ($0,s_{1}$), ($s_{1},s_{2}$), and ($s_{2},\tau_{S}$), when there are 2, 1, and 2 sequences, respectively, is $\exp\{-\frac{4M_{\mathrm{CB}}}{\theta_{B}}[2\left(s_{1}+\tau_{S}-s_{2}\right)+\left(s_{2}-s_{1}\right)]\}$. Contributions from species C,S,R similarly consist of coalescent and migration components.

Given the MSC-model and parameters, the gene trees are assumed to be independent among loci, so that the density for all gene trees is a product over the loci. Bayesian estimation of parameters in the model (Θ) involves averaging over all possible gene trees for all loci, which is achieved by the MCMC algorithm.

### Overview of MCMC Algorithms under the MSC-M Model.

We modified the MCMC proposals in ref. 19. We added sliding-window moves to update the migration rates (M) and the migration times on the gene trees. The subtree-pruning-and-regrafting (SPR) proposal for changing the gene-tree topology (19) was modified to accommodate migration events on the gene tree. We prune off a subtree and regraft it back to the gene-tree backbone by simulating the backward-in-time process of coalescent and migration using the current values of parameters (τ,θ,M). This proposal was originally implemented in migrate (35) and used in G-PhoCS (33). The proposal that updates population sizes (θ) remains unchanged. The mixing move is modified to rescale both migration times and coalescent times on the gene trees, together with the species divergence times (τ).

Note that in this paper, the MSC-M model (including the species phylogeny, the number of migration rate parameters and the direction and populations involved in migration) is fixed. We leave it to future work to implement cross-model algorithms to move in the space of MSC-M models.

We introduce two major changes to the rubber-band algorithm for updating species divergence times (19).

#### Composite-space algorithm for migration-rate parameters.

**Fig. 2.**
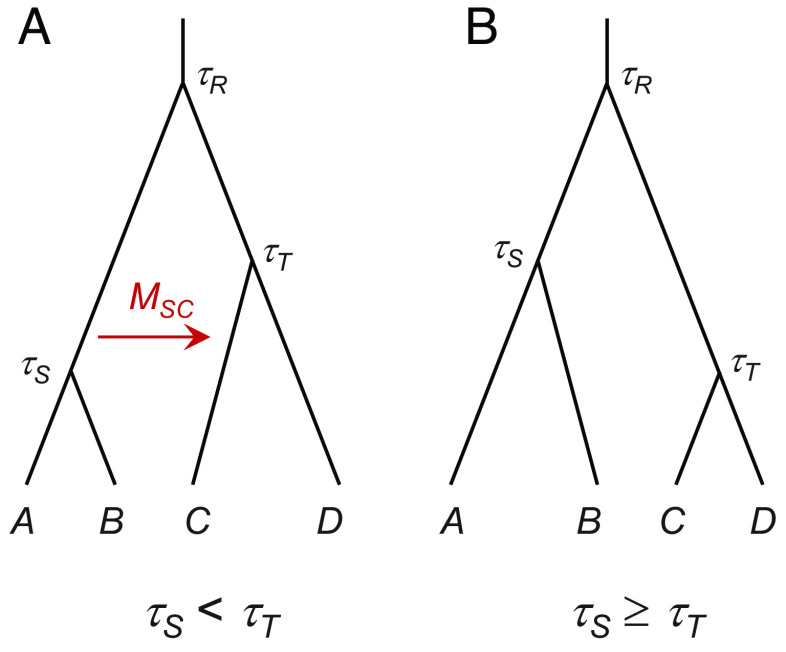
A balanced tree for four species with migration from S→C at the rate $M_{\mathrm{SC}}$. The migration rate parameter $M_{\mathrm{SC}}$ exists when (*A*) $\tau_{S}<\tau_{T}$ but disappears when (*B*) $\tau_{S}\geq \tau_{T}$.

First, we implement a trans-model MCMC algorithm to deal with the disappearance and reappearance of a migration-rate parameter when species divergence times change in the proposal. This is necessary because migration rate $M_{\mathrm{ij}}$ exists only when populations i and j are contemporary and changes in τ may cause contemporary populations to become noncontemporary or vice versa. As a result, a migration rate parameter which exists in the current model may not exist in the proposed model. Consider the species tree ((A,B),(C,D)) of Fig. 2, with migration rate $M_{\mathrm{SC}}$ from S to C. This exists only if $\tau_{S}<\tau_{T}$, when species S and C coexist during the time interval ($\tau_{S},\tau_{T}$). Otherwise, the two species are not contemporary, and $M_{\mathrm{SC}}$ is not a parameter in the model. The problem also appears when migration occurs in the opposite direction (C→S) or in both directions (C⇆S), and in large species trees with migration involving ancestral species.

In effect, the species tree represents two different models, depending on whether $\tau_{S}<\tau_{T}$, and the rubber-band algorithm for updating the species divergence time (τ), which is a within-model proposal under MSC with no gene flow, becomes trans-model and trans-dimensional under MSC-M. We combine different strategies of trans-dimensional MCMC algorithms (52–54), and implement a Carlin-Chib-Green-O’Hagan-metropolized (CCGOm) algorithm. See *SI Appendix, Methods and Materials* for details.

#### Extended rubber-band algorithm for the MSC-M model.

Second, we extend the rubber-band proposal (19) to accommodate migration events on gene trees. In G-PhoCS (33), a simple modification to the algorithm was introduced, whereby the rubber-band proposal is executed ignoring migration events but the proposal is abandoned if the proposed changes to coalescent times are in conflict with the migration events. Such a rejection approach may lead to poor mixing of the MCMC when there are many migration events on the gene trees as may happen at high migration rates or in large datasets with many loci. Here, we instead extend the rubber-band proposal to accommodate migration events explicitly, avoiding rejection. We identify a time interval ($\tau_{l},\tau_{u}$) affected by the proposal and populations in the time interval affected by migration, and then rescale affected migration times in the time interval according to the rubber-band algorithm (*SI Appendix*, Fig. S1). See *SI Appendix, Methods and Materials* for details.

We implemented the rejection algorithm (33) as well. Both the rejection and extended rubber-band algorithms showed good mixing when there are not many migration events on the gene trees across all loci, as in the analysis of small 100-loci datasets from the *Anopheles* genomes (Fig. 6*A*). However, in analyses of large datasets of *Anopheles* chromosomal arms, migration events were common, and the rejection algorithm mixed poorly due to frequent rejections, as seen from large differences among replicate runs (*SI Appendix*, Fig. S2). The extended rubber-band algorithm had better mixing properties for these data, producing consistent results among runs (*SI Appendix*, Fig. S3). Trace plots and posterior summaries for the two algorithms are shown for one dataset with 2223 coding loci on chromosome 2L1 (*SI Appendix*, Figs. S4 and S5).

### Bayesian Simulation to Validate the MCMC Algorithms.

Extensive tests have been conducted to validate our MCMC implementation (51). If no sequence data are used or if the likelihood is always set to 1, the MCMC algorithm should be sampling from the prior distribution, which is either known or analytically tractable. This test was effective in revealing most errors during the debugging stage.

Bayesian simulation was used to validate the algorithm more rigorously. Parameters are sampled from the prior and used to simulate each replicate dataset under the likelihood model, which is then analyzed using the same prior to generate the posterior. The average posterior over replicate datasets should then match the prior (55). For the test to be most effective, the datasets should be sufficiently large to allow the posterior for each dataset to be influenced by both the prior and the likelihood (data) and thus to differ among datasets, but small enough to allow inexpensive computation. Bayesian simulation tests both the simulation and inference components of the program.

The first MSC-M model used in our Bayesian simulation assumes one migration event from C→S on the species tree for three species of *SI Appendix*, Fig. S6*A*. Each dataset consists of L=250 loci, with S=4 sequences per species per locus, and N=500 sites in the sequence. We included G-PhoCS in the test. For bpp, we observed a close match between the prior and the average posterior for all eight parameters (*SI Appendix*, Fig. S6*B*). While the posterior differed among replicate datasets (*SI Appendix*, Fig. S6*C*), as the datasets were generated by using different parameter values and influenced by random sampling errors due to the finite data size, the average posterior over replicate datasets matched the priors, as expected.

G-PhoCS is an extension of an earlier version of bpp (19, 56) and uses a different parametrization of the MSC model, so that the two programs implement the same likelihood model, but use different priors. We sampled parameter values from the priors used in G-PhoCS to simulate replicate datasets, and analyzed them using G-PhoCS. See *SI Appendix, Methods and Materials*. There was a close match between the prior and the average posterior for all parameters (*SI Appendix*, Fig. S6 *D* and *E*).

The second MSC-M model used was a saturated model for three species with eight migration rates (*SI Appendix*, Fig. S7*A*). This is parameter-rich, and the rates for ancestral migration ($M_{\mathrm{SC}}$ and $M_{\mathrm{CS}}$) are particularly challenging to estimate. We used L=1,000 loci, with S=4 sequences per species per locus and N=500 sites in the sequence. For bpp, we observed a close match between the prior and the average posterior for all 15 parameters (*SI Appendix*, Fig. S7).

There was great disparity in information content among the parameters in the model: species divergence times (τ) and population sizes (θ) were very well estimated with sharp posteriors, whereas the posterior of migration rates was diffuse (*SI Appendix*, Fig. S7*C*). The six migration rates involving extant species were more precisely estimated than the two migration rates involving the ancestral species S. Also $M_{\mathrm{CS}}$ was better estimated than $M_{\mathrm{SC}}$, with sharper posteriors, apparently because twice as many sequences (from A and B) reach node S in the species tree as sequences from C reaching $\tau_{S}$ in lineage C, when we trace the genealogy of the sampled sequences backward in time. While the simulation validates the program, datasets with 1,000 loci were not informative enough to estimate all eight migration rates with high precision. We did not use G-PhoCS in this test under the saturated model due to the computational cost, but instead analyzed one dataset of 2,000 loci for comparison with bpp (see below).

### Simulation under a Saturated MSC-M Model for Three Species.

**Fig. 3.**
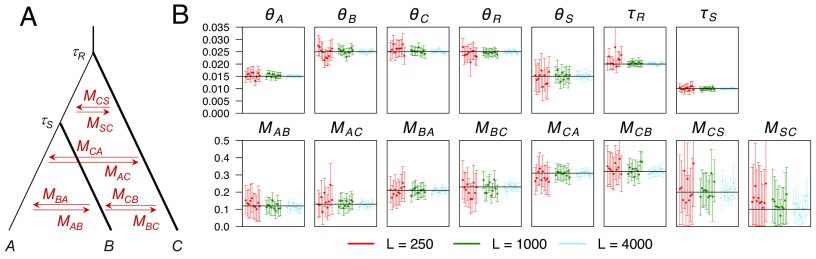
(*A*) The saturated migration model for three species with eight migration rates, used to simulate data for analysis by bpp. The parameters used are $\tau_{R}=0.02$, $\tau_{S}=0.01$, $\theta_{A}=\theta_{S}=0.015$, and $\theta_{B}=\theta_{C}=\theta_{R}=0.025$. The eight migration rates are $M_{\mathrm{AB}}=0.12$, $M_{\mathrm{BA}}=0.21$, $M_{\mathrm{AC}}=0.13$, $M_{\mathrm{CA}}=0.31$, $M_{\mathrm{BC}}=0.23$, $M_{\mathrm{CB}}=0.32$, $M_{\mathrm{CS}}=0.2$, and $M_{\mathrm{SC}}=0.1$. (*B*) Posterior means and 95% HPD CIs of parameters in 10 replicate datasets of different sizes, with L=250, 1,000, 4,000 loci. Horizontal lines represent the true parameter values. A large dataset of L= 16,000 loci is analyzed in *SI Appendix*, Table S1.

To examine the statistical performance of our method, we simulated replicate datasets under the saturated MSC-M model of figure 3a using increasing numbers of loci (L=250, 1,000 and 4,000 loci) with S=4 sequences per species per locus. We have two objectives: i) to confirm the correctness of our implementation—indicated by the convergence of Bayesian estimates of parameters to their true values with increasing numbers of loci, and ii) to address the practical question of whether typical genomic datasets contain enough information to allow reliable estimation of the eight migration rates (and other parameters in the model).

The 95% highest probability density (HPD) credible intervals (CIs) for parameters included the true values and became narrower with the increase of data size (Fig. 3*B*), as expected from the consistency of Bayesian estimation. The divergence times (τ) and population sizes (θ) were very well estimated, but the migration rates (especially $M_{\mathrm{CS}}$ and $M_{\mathrm{SC}}$) involved large uncertainties even with L=4,000 loci. For further confirmation, we simulated a large dataset of 16,000 loci and found that the estimates of migration rates became much more precise (*SI Appendix*, Table S1). Overall, the results (Fig. 3*B* and *SI Appendix*, Table S1) suggest the correctness of our MCMC implementation. They also suggest that genomic datasets with >10${}^{4}$ loci may contain sufficient information to allow precise and accurate estimation of all migration rates in the saturated model (as well as species divergence times and population sizes).

We analyzed a dataset of 2,000 loci simulated under the saturated model extensively using bpp and G-PhoCS (*SI Appendix*, Table S1). Estimates of population sizes were similar between the two programs, but large differences existed in estimates of species divergence times and migration rates. Relative to bpp estimates and to true parameter values, G-PhoCS estimates of $\tau_{R}$ and $\tau_{S}$, and of $M_{\mathrm{SC}}$ and $M_{\mathrm{CS}}$, were too small while those of $\theta_{S}$ were too large (*SI Appendix*, Table S1). The differences do not appear to be due to the minor differences between the priors used by the two programs or to mixing issues of the MCMC algorithms in G-PhoCS, and instead suggest that implementation of the MSC-M model in G-PhoCS was not correct.

### Simulation under an MSC-M Model with Three Species and Two Migration Rates: Comparison with IMA3.

**Fig. 4.**
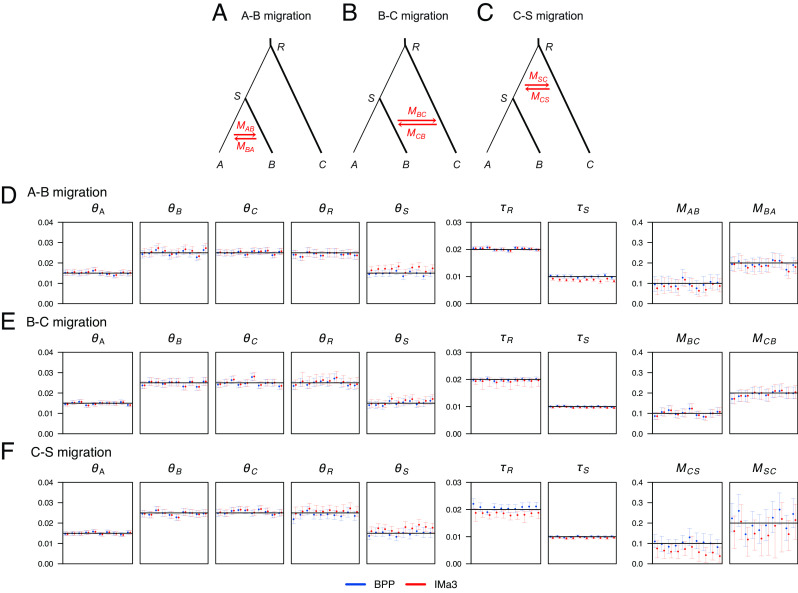
(*A*–*C*) Three MSC-M models for three species with (*A*) A–B migration (between sister species), (*B*) B–C migration (between nonsister species), and (*C*) C–S migration (between sister species and involving one ancestor) used for simulating data, for analysis using bpp and IMA3. The parameters used are $\tau_{R}=0.02$, $\tau_{S}=0.01$, $\theta_{A}=\theta_{S}=0.015$, $\theta_{B}=\theta_{C}=\theta_{R}=0.025$, with migration rates M = 0.1 in one direction and 0.2 in the opposite direction. (*D*–*F*) Posterior means and 95% CIs for the nine parameters in the model obtained using bpp (blue) and IMA3 (red) in 10 replicate datasets. Black horizontal lines represent true values. IMA3 uses mutation rate per locus, not per site; estimates from IMA3 are thus divided by the sequence length. IMA3 outputs the population migration rate 2M; the estimates are divided by 2.

We compared bpp with IMa3 by analyzing datasets simulated under the MSC-M models of Fig. 4*A*–*C* on a species tree for three species. As IMa3 assumes bidirectional migration, each model has a pair of migration rates. We generated 10 replicate datasets under the JC mutation model, each consisting of L=500 loci, S=4 sequences per species per locus, and N=500 sites in the sequence. The results are presented in Fig. 4*D*–*F*.

The bpp results are as expected, with most of the 95% CIs bracketing the true values (Fig. 4*D*–*F*). Migration rates between sister lineages are harder to estimate than between nonsister lineages, and those involving ancestral species are harder than those between extant species. Indeed, the estimates in model B (B–C migration) had the narrowest CIs while those in model C (S–C migration) had the widest (Fig. 4*D*–*F*).

Overall, IMa3 produced similar posterior results to bpp (Fig. 4*D*–*F*). The correlation between the point estimates (posterior means) from the two programs was 0.997, 1.000, and 0.984, for models A, B, and C, respectively. Under models A and B, the estimates were particularly similar, although IMa3 appeared to overestimate $\theta_{S}$ and underestimate $\tau_{S}$ slightly. Under the more challenging model C (C-S migration), most θ parameters were well estimated by IMa3, but $\tau_{R}$ was underestimated with the CIs excluding the true value, and the migration rates were underestimated. These “biases” do not appear to be due to mixing issues or to reflect the impact of the prior and instead indicate implementation problems. Note that the prior mean for $\tau_{R}$ was 0.024, larger than the true value 0.02, while the prior means of $M_{\mathrm{CS}}$ and $M_{\mathrm{SC}}$ were equal to the true values (*SI Appendix, Methods and Materials*).

We conclude that IMa3 and bpp produced very similar results under simple migration models with migration involving extant species (Fig. 4 *D* and *E*), while bpp was more reliable under challenging models with migration involving ancestral species (Fig. 4*F*). Also bpp had a computational advantage in large datasets. Note that IMa3 includes cross-model moves that change the species phylogeny (57), whereas the model is fixed in bpp and G-PhoCS.

### Simulation under the Stepping-stone and Island Models: Comparison with MIGRATE.

Our bpp implementation of the MSC-M model may also be used to perform inference under classical population genetic models of subdivision with migration. Models of population subdivision are typically applied to different populations of the same species and do not incorporate a phylogeny for the populations. They may be viewed as a special case of the MSC-M model with ancient species divergences (e.g., with τ→∞). In other words, if the probability is essentially 100% that all sequences sampled from the extant species have coalesced or reached their most recent common ancestor (MRCA) before the time of the most recent species divergence (with time running backward), the two classes of models will be equivalent. The MSC-M model is thus an extension of the structured coalescent model to incorporate a species/population phylogeny.

**Fig. 5.**
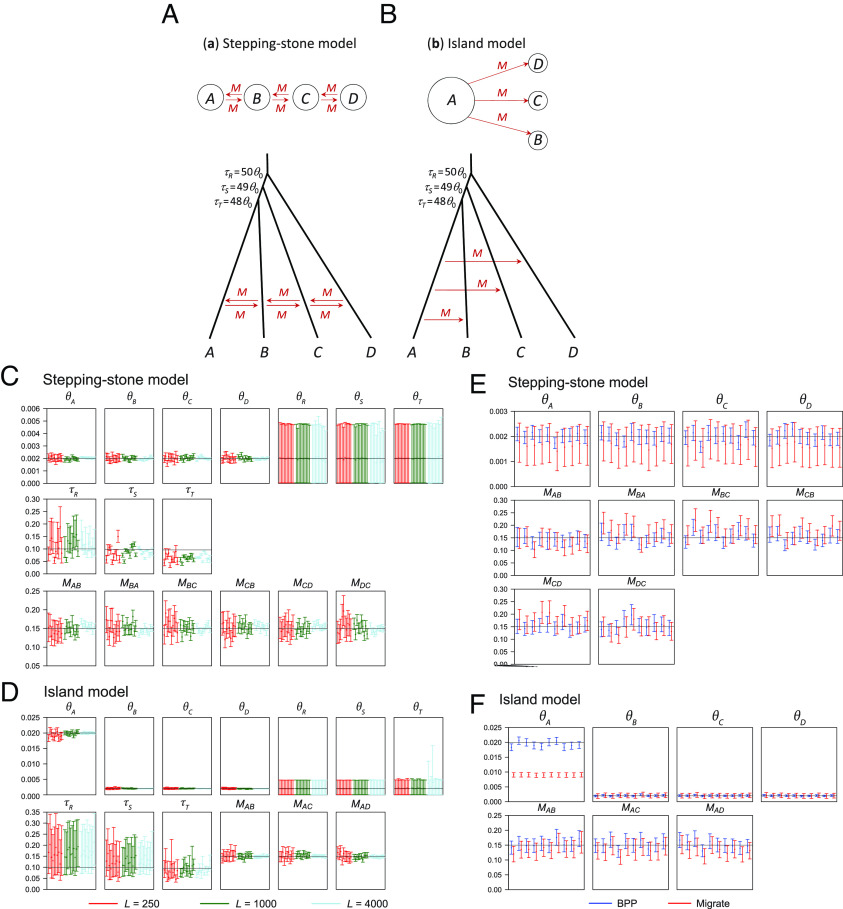
(*A*) Stepping-stone and (*B*) island models used for simulating data, for analysis by using bpp and migrate. The population genetic models (*Top*) are approximated by the MSC-M models with very large divergence times (*Bottom*). All population sizes are $\theta_{0}=0.002$ except that $\theta_{A}=10\theta_{0}$ in the island model. The migration rate was M=0.15 migrants per generation. (*C* and *D*) Posterior means and 95% HPD CIs of parameters in bpp analyses of 10 replicate datasets (each of L=250, 1,000, or 4,000 loci) simulated under the models. The horizontal lines represent the true values. (*E* and *F*) The datasets of 250 loci are also analyzed using migrate (red), in comparison with bpp (blue). migrate uses the mutation-scaled migration rate, $\varpi_{\mathrm{ij}}=4M_{\mathrm{ij}}/\theta_{j}$ in the notation here; this is transformed to ${\hat{M}}_{\mathrm{ij}}={\hat{\varpi }}_{\mathrm{ij}}{\hat{\theta }}_{j}/4$ by using the posterior mean ${\hat{\theta }}_{j}$.

We conducted a simulation under the stepping-stone and island models (Fig. 5 *A* and *B*) and analyzed the data using both bpp and migrate (58). Very large divergence times were used to simulate data so that sequences sampled from all species at any locus coalesce with near certainty before reaching any species divergence event. Under these conditions, we expect bpp estimates of species divergence times to be very large (and uncertain) and the posterior distribution of ancestral population sizes to match the prior, while migration rates and populations sizes for extant species should be reliably estimated.

Parameter estimates from bpp under the stepping-stone model are summarized in Fig. 5*C* and *SI Appendix*, Table S2. As expected, the posteriors for θ for ancestral populations were nearly the same as the prior. There appeared to be a minimal amount of information about species divergence times (τ) as the CIs became narrower with more loci. The estimates were smaller than the true values, due to the influence of the priors. Our interest is in estimation of population sizes for extant species ($\theta_{A},\theta_{B},\theta_{C},\theta_{D}$) and migration rates (M). These were well estimated, with the posterior means fluctuating around the true values (Fig. 5*C*) and with the CI becoming narrower with an increase in the number of loci (L). The 95% HPD CIs matched the large-sample expectation that quadrupling the number of loci halves the CI width (*SI Appendix*, Table S2). We note that estimates of migration rates involved considerable uncertainty even in large datasets of L=4,000 loci.

The results under the island model (Fig. 5*D* and *SI Appendix*, Table S2) similarly suggest little information in the data concerning the species divergence times ($\tau_{R},\tau_{S},\tau_{T}$) and ancestral population sizes ($\theta_{R},\theta_{S},\theta_{T}$), but population sizes for extant species ($\theta_{A},\theta_{B},\theta_{C},\theta_{D}$) were well estimated, as were the migration rates ($M_{\mathrm{AB}},M_{\mathrm{AC}},M_{\mathrm{AD}}$).

For comparison, we used migrate (58) to analyze the small datasets of L=250 loci. For the stepping-stone model, migrate estimates 4 θs and 6 M parameters, while bpp estimates 7 θs, 3 τs, and 6 M rates. We focus on the shared parameters. While both programs use the same definitions of divergence times (τ) and population sizes (θ), migrate uses the mutation-scaled migration rate, which is $\varpi_{\mathrm{ij}}=4M_{\mathrm{ij}}/\theta_{j}$ in the notation of this paper. We assigned gamma priors on $\varpi_{\mathrm{ij}}$ similar to priors used in the bpp analysis (*SI Appendix, Methods and Materials*). For easy comparison with bpp, we then converted the migrate estimates of migration rates into ${\hat{M}}_{\mathrm{ij}}={\hat{\varpi }}_{\mathrm{ij}}{\hat{\theta }}_{j}/4$, using the posterior mean ${\hat{\theta }}_{j}$. Estimates of migration rates under the stepping-stone model were very similar between the two programs (Fig. 5*E*), although the migrate estimates had slightly wider CIs. migrate estimates of θ for extant species were too small with wide CIs, compared with the bpp estimates and with the true values. For the island model, migrate estimates 4 θs and 3 migration rates (M), while bpp estimates 7 θs, 3 τs, and 3 migration rates. Estimates of migration rates were similar between the two programs (Fig. 5*F*). migrate estimates of $\theta_{B}$, $\theta_{C}$, and $\theta_{D}$ were centered around the true values but had wider CIs than the bpp estimates. migrate estimates of $\theta_{A}$ were much too small relative to bpp estimates or the true values (Fig. 5*F*).

The results suggest problems with the migrate implementation of the stepping-stone and island models. migrate does not write the sampled parameter values into a disk file but collects them into pre-defined bins based on the priors, and the resulting histograms are then smoothed to estimate the posterior probability densities and to calculate the posterior means and HPD intervals. This may cause inaccurate posterior summaries if the number of bins is small and if the prior and the posterior are very different. We used a large number of bins (3,000 or 10,000) and found the results to be stable. Similarly, we rule out issues in kernel density smoothing and differences in reparametrizations and the prior as the main reasons for the differences, because the estimated migration rates were similar between the two programs and the large differences were in the population sizes for extant species ($\theta_{A}$–$\theta_{D}$), which should be easy to estimate (59).

### Analysis of Genomic Data from the A. gambiae Mosquitoes.

**Fig. 6.**
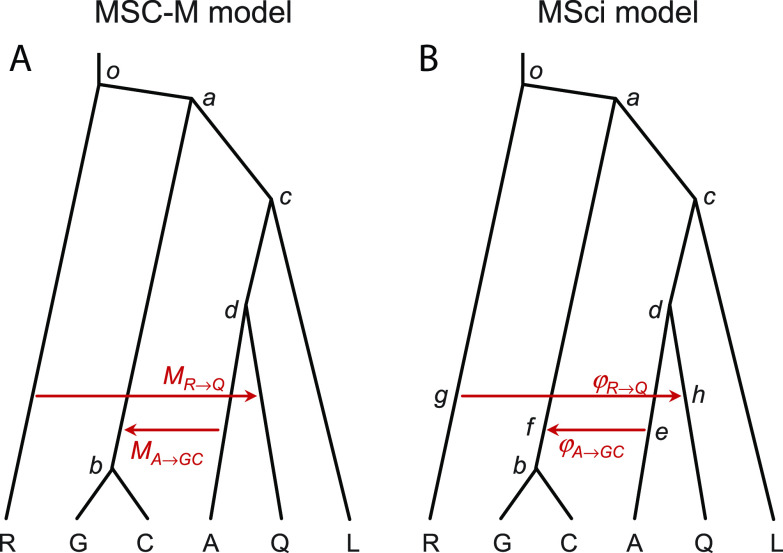
(*A*) MSC-M and (*B*) MSC-I models for six species of African mosquitoes in the *A. gambiae* species complex: *A. gambiae* (G), *A. coluzzii* (C), *A. arabiensis* (A), *A. melas* (L), *A. merus* (R), and *A. quadriannulatus* (Q).

We used the MSC-M model as well as the MSC-I model (21) to analyze the coding and noncoding data from six species of African mosquitoes in the *A. gambiae* species complex. The MSC-I model constructed in ref. 43 includes two introgression events (Fig. 6*B*), which are replaced by migration to form an MSC-M model (Fig. 6*A*).

#### Bayesian test of gene flow using blocks of 100 loci.

**Fig. 7.**
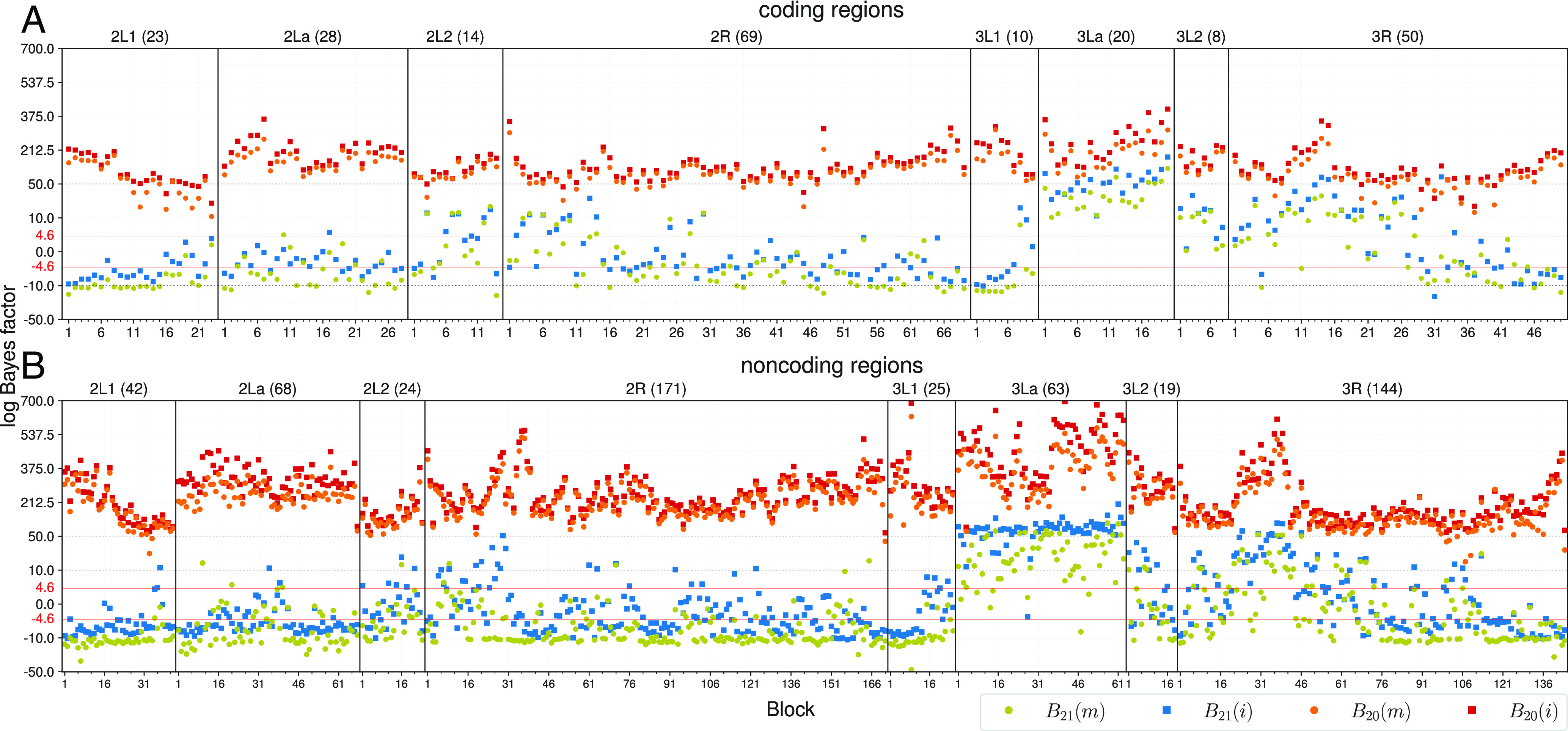
The logarithm of the Bayes factor for testing gene flow obtained from bpp analysis of the 100-loci blocks of the (*A*) coding and (*B*) noncoding data from the *Anopheles* mosquitoes (Fig. 6*A*), calculated using thermodynamic integration with Gaussian quadrature (42). Gene flow is accounted for using either the introgression model (i for MSC-I) or the migration model (m for MSC-M). Model $H_{0}$ is the MSC model with no gene flow. Model $H_{1}$ assumes the A→GC gene flow, with rate $M_{A\to GC}$ under MSC-M or $\varphi_{A\to GC}$ under MSC-I. Model $H_{2}$ accommodates both gene-flow events, with rates $M_{A\to GC}$ and $M_{R\to Q}$ under MSC-M or $\varphi_{A\to GC}$ and $\varphi_{R\to Q}$ under MSC-I. Bayes factor $B_{20}$ measures the support for $H_{2}$ over $H_{0}$, while $B_{21}$ measures the support for $H_{2}$ over $H_{1}$. The test is significant when |logB|>4.6 (i.e., if B>100 or B<0.01). For example, $\log B_{21}$(m) <−4.6 means that the data strongly support the one-rate model with the A→GC migration (with rate $M_{A\to GC}$) over the two-rates model with both the A→GC and the R→Q migrations. Different scales are used for the y-axis over the intervals (−50,−10),(−10,10),(10,50),(50,700).

First, we analyzed blocks of 100 loci to test for the presence of gene flow (Fig. 7). We calculated Bayes factors using thermodynamic integration with Gaussian quadrature (42, 60) to compare three models of gene flow:

$H_{0}$: MSC with no gene flow;
$H_{1}$: MSC + A→GC gene flow;
$H_{2}$: MSC + A→GC and R→Q gene flow.

$B_{20}$(m) and $B_{20}$(i) are Bayes factors in support of $H_{2}$ over $H_{0}$, with migration under the MSC-M and introgression under the MSC-I, respectively (Fig. 7). We considered B>100 or B<0.01 (or |logB|>4.6) to be “significant”; this is similar to a 1% “significance level.” There is strong evidence for gene flow as $B_{20}>100$ in every block and under both MSC-M and MSC-I models. In most blocks, $B_{20}$(i) $>B_{20}$(m), with the introgression model fitting the data better than the migration model.

$B_{21}$ is the Bayes factor in support of $H_{2}$ over $H_{1}$ and tests for R→Q gene flow when the model already accommodates A→GC gene flow. This test was significant for most 100-loci blocks in the 3La inversion region and the 3L2 region, but not in most blocks on chromosome 2. The pattern was similar between the MSC-M and MSC-I models. In most blocks, $B_{21}$(i) $>B_{21}$(m), with stronger evidence for R→Q gene flow under MSC-I than under MSC-M. Indeed, $B_{21}$(m) <0.01 in many blocks on chromosome 2, with strong support for the null model of no R→Q gene flow. Note that Bayesian model selection may strongly support the null model, unlike the likelihood ratio test, which may fail to reject the null but never strongly supports it. In sum, both MSC-I and MSC-M models strongly support A→GC gene flow, with evidence for R→Q gene flow mostly involving chromosome 3.

#### Variable rates of gene flow across blocks of 100 loci.

Next, we examine the estimated migration rates. $M_{A\to GC}$ and $M_{R\to Q}$ under MSC-M varied among the blocks or across the genome, as did the corresponding introgression probabilities under MSC-I (*SI Appendix*, Fig. S8). There was overall consistency between the coding and noncoding data. $M_{A\to GC}$ was high (>0.1) in most blocks except those on 2L1 and 3L1. $M_{R\to Q}$ was low for most blocks except those from 3La and 3R. Estimates of $\varphi_{A\to GC}$ and $\varphi_{R\to Q}$ under MSC-I were similar to those in figure 6 of ref. 21, where inverse-gamma priors were used for τ and θ. Here, we used gamma priors and assumed the same θ before and after each introgression event for each branch on the species tree. Both introgression probabilities varied considerably across the genome, with $\varphi_{A\to GC}\approx 100\%$ in most blocks.

As the evidence for A→GC gene flow was overwhelming, we focus on the R→Q gene flow under $H_{2}$. There was no good correspondence between the estimated migration rate ${\hat{M}}_{R\to Q}$ and introgression probability ${\hat{\varphi }}_{R\to Q}$ (*SI Appendix*, Fig. S9). The MSC-M model predicts that the probability that any sequence from Q is traced to population R to be (26)

[4] $$\begin{array}{c} \varphi_{0,RQ}=1-e^{-4M_{\mathrm{RQ}}\tau_{d}/\theta_{Q}}. \end{array}$$

If MSC-M is the true model, Eq. **4** is expected to give an upper bound for the estimate when the data are analyzed under MSC-I: $\hat{\varphi }<\varphi_{0}$ (26): figure 1]. However, our estimates displayed very poor matches between $\varphi_{0,RQ}$ under MSC-M and ${\hat{\varphi }}_{\mathrm{RQ}}$ under MSC-I (*SI Appendix*, Fig. S10), even though estimates of $\tau_{d}$ and $\theta_{Q}$ were very similar under the two models (*SI Appendix*, Fig. S11). The reasons for this lack of correspondence are unclear. One possibility is that the MSC-M model (Fig. 6*A*) is a poor fit to the genomic data: The rate of gene flow might vary over time, but on average, the MSC-I model assuming a pulse of gene flow was closer to reality than the MSC-M model assuming a constant rate over the whole time period. In other words, introgression events detected from the genome data may be largely historical. Estimated frequencies of *A. gambiae*×*A. arabiensis* F${}_{1}$ hybrids for modern species where the two species are sympatric were low, around 0.15–0.22% (61) and references therein].

While the MSC-M and MSC-I models make very different assumptions about the mode of gene flow, they produced highly similar estimates of species divergence times ($\tau_{o},\tau_{a},\tau_{c},\tau_{d}$) (*SI Appendix*, Fig. S11). The results are consistent with the simulation study of ref. 26, which found that species divergence times were well estimated when the mode of gene flow was misspecified. In contrast, the MSC model of no gene flow seriously underestimated divergence times, as found in simulations (62).

#### Parameter estimation for chromosomal arms.

Finally, we analyzed all loci for each chromosomal arm as one dataset. The posterior means and 95% HPD CIs for the migration rates are in Table 1, while species divergence times and migration rates are summarized in *SI Appendix*, Fig. S12 *A*–*D*. We have used those large datasets to illustrate mixing properties of the rejection and extended rubber-band algorithms in *SI Appendix*, Figs. S2–S5.

**Table 1.** Bayesian estimates of migration rates (M) and of introgression probabilities (φ) from the *Anopheles* genomic data (Fig. 6)

		*A. arabiensis*→*A. gambiae* + *A. coluzzii*		*A. merus* → *A. quadriannulatus*	
Dataset	Loci	${\hat{M}}_{A\to GC}$	${\hat{\varphi }}_{A\to GC}$	${\hat{M}}_{R\to Q}$	${\hat{\varphi }}_{R\to Q}$
2L1 coding	2,223	0.404 (0.366, 0.443)	0.955 (0.933, 0.975)	0.002 (0.000, 0.003)	0.029 (0.016, 0.043)
2L1 noncoding	4,133	0.311 (0.293, 0.329)	0.963 (0.950, 0.975)	0.000 (0.000, 0.001)	0.016 (0.008, 0.024)
2La coding	2,776	2.451 (2.122, 2.789)	0.791 (0.768, 0.813)	0.005 (0.002, 0.008)	0.038 (0.006, 0.074)
2La noncoding	6,732	2.289 (2.116, 2.466)	0.696 (0.684, 0.708)	0.001 (0.000, 0.001)	0.015 (0.007, 0.022)
2L2 coding	1,362	1.053 (0.874, 1.233)	0.879 (0.847, 0.910)	0.030 (0.020, 0.041)	0.180 (0.134, 0.228)
2L2 noncoding	2,330	0.618 (0.565, 0.672)	0.955 (0.936, 0.974)	0.008 (0.005, 0.012)	0.074 (0.056, 0.091)
2R coding	6,849	0.909 (0.844, 0.977)	0.971 (0.962, 0.979)	0.010 (0.008, 0.013)	0.074 (0.063, 0.085)
2R noncoding	17,027	0.739 (0.712, 0.771)	0.978 (0.974, 0.982)	0.003 (0.002, 0.003)	0.047 (0.042, 0.052)
3L1 coding	983	0.215 (0.189, 0.242)	0.967 (0.948, 0.985)	0.003 (0.001, 0.006)	0.058 (0.034, 0.084)
3L1 noncoding	2,496	0.234 (0.218, 0.249)	0.976 (0.965, 0.987)	0.001 (0.000, 0.002)	0.033 (0.019, 0.047)
3La coding	1,998	1.708 (1.454, 1.971)	0.929 (0.914, 0.945)	0.153 (0.131, 0.176)	0.600 (0.569, 0.631)
3La noncoding	6,208	1.399 (1.299, 1.498)	0.973 (0.968, 0.978)	0.083 (0.077, 0.090)	0.619 (0.604, 0.634)
3L2 coding	764	1.577 (1.261, 1.913)	0.923 (0.896, 0.948)	0.043 (0.029, 0.057)	0.306 (0.234, 0.378)
3L2 noncoding	1,823	2.003 (1.700, 2.300)	0.951 (0.937, 0.964)	0.012 (0.008, 0.017)	0.161 (0.131, 0.192)
3R coding	4,977	0.788 (0.727, 0.853)	0.939 (0.927, 0.952)	0.028 (0.023, 0.034)	0.168 (0.149, 0.188)
3R noncoding	14,323	0.636 (0.612, 0.663)	0.959 (0.953, 0.965)	0.012 (0.011, 0.014)	0.103 (0.095, 0.111)

Similarly to the analyses of the 100-loci blocks, migration rates and introgression probabilities varied considerably among chromosomal arms. ${\hat{M}}_{A\to GC}$ was high for all chromosomal arms, with the smallest being ∼0.2 for 3L1 (coding), while ${\hat{\varphi }}_{A\to GC}>0.9$ for all chromosomal arms except 2La (coding and noncoding) and 2L2 (coding). Note that the A→GC gene flow is so prevalent for the autosomes that the predominant autosomal gene tree has a different topology from the species phylogeny (2, 43). The R→Q gene flow mostly affected 3La and 3L2, while 2L2 and 3R were affected to a lesser extent.

Estimates of species divergence times were highly consistent among the chromosomal arms and between the coding and noncoding data (*SI Appendix*, Figs. S12 *A*–*D* and S13). Most estimates (in particular, those from the noncoding data) had tight CIs because of the large data sizes, although $\tau_{a}$ had wider CI bars as the estimates were affected by the estimated rate of A→GC gene flow.

## Discussion

Models of population subdivision and migration developed in population genetics are special cases of the MSC-M model. The stepping-stone and island models (Fig. 5 *A* and *B*) are instances of the MSC-M model with population divergence times approaching ∞. Our results (Fig. 5) suggest that bpp is an efficient and reliable implementation of such population genetic models, allowing them to be fitted to genomic data. We note that other specialized models may also be special cases of the MSC-M model. For example, the isolation-with-initial-migration (IIM) model assumes that migration occurred initially after species divergence but stopped at a certain time point, for example, when reproductive isolation is fully established (63–65). In the secondary contact (SC) model (65), two species initially experienced complete isolation after divergence but came into contact at a certain time point, with subsequent ongoing migration. Both IIM and SC models can be fitted to genomic data as instances of the MSC-M model by including an unsampled ghost species (26).

The MSC-M model extends models of population subdivision to incorporate a population/species phylogeny (16, 66, 67). Besides improving the biological realism of the model, this extension also opens up opportunities for addressing many important questions in evolutionary biology, such as detecting gene flow during and after speciation, delineating species boundaries in the presence of gene flow, inferring historical demographic changes or estimating population sizes for extinct ancestral species, detecting gene flow from extinct species that may and may not have extant descendents (16). Likelihood-based implementations of the MSC-M model have involved heavy computation and are impractical for genome-scale data of thousands of loci, although large genomic datasets are routinely generated and are indeed necessary for precise and accurate estimation of the rate of gene flow. Furthermore, it is challenging to implement the model correctly: While both G-PhoCS (33) and migrate (58) have undergone extensive testing and validation, our simulation and test suggest errors in implementation.

Here, our stringent tests using Bayesian simulation and conventional methods for evaluating mixing and statistical properties of estimates suggest that the MCMC algorithms in bpp are correctly sampling from the posterior and that our algorithms have improved mixing. While it is computationally demanding, bpp has been applied to datasets of >10,000 loci (Table 1, *SI Appendix*, Table S1). We suggest that our implementation of the MSC-M model in bpp provides a useful tool for comparative analysis of genomic data to infer gene flow between divergent species or populations and a platform for engineering further algorithmic improvements.

## Materials and Methods

Detailed descriptions of algorithms, simulation conditions, and analyses of simulated and *Anopheles* data are in online *SI Appendix, Supplemental Text*.

## Supplementary Material

Appendix 01 (PDF)

## Data Availability

The *Anopheles* genomic data are available at http://abacus.gene.ucl.ac.uk/ziheng/data/AnophelesData2020.tgz (68). The MCMC algorithms are implemented in bpp, available under GPL3 at https://github.com/bpp/ (69).
