# Supplementary material for: Efficient Bayesian inference under the multispecies coalescent with migration
Source: Proc Natl Acad Sci U S A. 2023 Oct 23;120(44):e2310708120. doi: 10.1073/pnas.2310708120 (PMC10622872; doi:10.1073/pnas.2310708120)
Supplement: Supplementary file 1 — Appendix 01 (PDF) [file pnas.2310708120.sapp.pdf]

Supporting Information for

Efficient Bayesian inference under the multispecies coalescent with migration

Tomáš Flouri, Xiyun Jiao, Jun Huang, Bruce Rannala and Ziheng Yang

Corresponding Author name.
E-mail: bhrannala@ucdavis.edu, z.yang@ucl.ac.uk

This PDF file includes:

Supporting text
Figs. S1 to S13
Tables S1 to S2
SI References

Supporting Information Text

SI text 1. Composite-space algorithm for migration-rate parameters. Changes to species divergence times during the MCMC may cause contemporary species to become non-contemporary. As a result, a migration rate parameter which exists in the current model may not exist in the proposed model. Consider the species tree $((A, B)(C, D))$ of figure 2, with migration rate M_{SC} from S to C. This exists only if $\tau_S < \tau_T$, when species S and C coexist during the time interval (τ_S, τ_T). Otherwise the two species are not contemporary, and M_{SC} is not a parameter in the model. The problem also appears when migration is in the opposite direction ($C \rightarrow S$) or in both directions ($C \leftrightarrows S$), and in large species trees with migration involving ancestral species. The problem appears to have been ignored in implementation of G-PhoCS (1).

In effect, the species tree represents two different models, depending on whether $\tau_S < \tau_T$, and the rubber-band algorithm for updating the species divergence time (τ), which is a within-model proposal under MSC with no gene flow, becomes trans-model and trans-dimensional under MSC-M. Several approaches have been suggested to deal with dimension changes in MCMC. In the product-space approach (2), all parameters from all models are collected into a vector. When the MCMC is visiting one model, parameters of all other models are called *pseudo-parameters* and are assigned *pseudo-priors*. A Gibbs sampler is used to update all parameters, including the pseudo-parameters. Use of good pseudo-priors that are similar to the posterior of the parameters is noted to be critical to the success of the algorithm, and pilot runs are used to approximate the posterior of the parameters (2). The well-known reversible-jump MCMC (rjMCMC) (3) emphasizes dimension matching between models, but a drawback is the difficulty of proposing values of parameters for the new model that do not exist in the current model; poor choices often lead to rejection of the proposal. The composite-space construction (4) allows arbitrary overlap of parameters between models.

Here we combine ideas in those frameworks (2, 4, 5), and implement a Carlin-Chib-Green-O'Hagan-metropolized (CCGOm) algorithm. Common parameters unaffected by a change to τ are mapped between the models, while a migration rate that ceases to exist due to a change in the relative order of two species divergence times (τ_S and τ_T in the example) is treated as a pseudo-parameter with a pseudo-prior assigned, as in ref. (2). However, unlike ref. (2), we do not update pseudo-parameters that are inactive in the current model, as such updates are unnecessary (5). We also use a Metropolis step of acceptance/rejection to move between the two models, instead of the Gibbs sampler (2). This has two advantages. First it does not require the conditional distribution, which is intractable. Second, the Metropolis step makes the proposal flexible and the choice of the pseudo-prior less critical to efficient mixing of the algorithm (even though it is still advantageous to choose the pseudo-prior to be close to the posterior). Furthermore, the algorithm may be preferable to rjMCMC in that the migration-rate parameter for the new model is not generated anew at each time but takes the value last visited when the chain was in that model.

Here we describe the algorithm for the particular case of updating τ_S or τ_T on the species tree $((A, B), (C, D))$, to deal with the disappearance and reappearance of migration rate M_{SC}. Larger species trees or more complex migration models are treated similarly. For simplicity, we write the migration rate as $M_{SC} \equiv M_1$ and let $\boldsymbol{M}_{-1}$ be the other migration rates in the model not affected by the proposal to change τ, with $\boldsymbol{M} = (M_1, \boldsymbol{M}_{-1})$. The species tree represents two models with different numbers of parameters: $H_0 : \tau_S \geq \tau_T$, in which M_1 does not exist, and $H_1 : \tau_S < \tau_T$, which includes M_1 (fig. 2). Let $p_1(M_1)$ be the prior of M_1 under H_1 and $p_1(M_1|X)$ the posterior. M_1 is a pseudo-parameter in H_0. Let $p_0^*(M_1)$ be the pseudo-prior in H_0 so that the posterior $p_0^*(M_1|X) = p_0^*(M_1)$. Suppose the Markov chain is currently in H_0 with $\tau_S \geq \tau_T$, and the rubber-band proposal changes one of the species divergence times such that $\tau'_S < \tau'_T$, so that the proposed model is H_1. When we move from H_0 to H_1, we use the value of M_1 last visited when the chain was in H_1. The proposal also changes the gene trees from G to G'. The proposal from H_0 to H_1 is then accepted with probability $\min\{1, A_{01}\}$, where

$$A_{01} = \frac{q(\boldsymbol{\tau}, G|\boldsymbol{\tau}', G')}{q(\boldsymbol{\tau}', G'|\boldsymbol{\tau}, G)} \times \frac{p_1(\boldsymbol{\tau}', \boldsymbol{\theta}, \boldsymbol{M}_{-1}|H_1)}{p_0(\boldsymbol{\tau}, \boldsymbol{\theta}, \boldsymbol{M}_{-1}|H_0)} \times \frac{p_1(M_1)}{p_0^*(M_1)} \times \frac{p(G'|H_1, \boldsymbol{\tau}', \boldsymbol{\theta}, \boldsymbol{M}_{-1}, M_1)}{p(G|H_0, \boldsymbol{\tau}, \boldsymbol{\theta}, \boldsymbol{M}_{-1})} \times \frac{p(X|G')}{p(X|G)}, \qquad [1]$$

where $\frac{q(\tau, G|\tau', G')}{q(\tau', G'|\tau, G)}$ is the Hastings ratio of the rubber-band move (6). The pseudo-prior $p_0^*(M_1)$ does not influence the posterior for parameters of interest, and is a computational device that affects MCMC mixing. While it is flexible, it should be chosen to resemble the posterior $p_1(M_1|X)$ under H_1.

The reverse rubber-band move from H_1 to H_0 is accepted with probability $\min\{1, A_{10}\}$, with $A_{10} = \frac{1}{A_{01}}$.

Note that in H_0, M_1 is a pseudo-parameter. When the MCMC sample is processed to summarize the posterior, it is incorrect to summarize all samples of M_1 without checking whether $\tau_S < \tau_T$. Samples of M_1 conditional on $H_0 : \tau_S \geq \tau_T$ are from the pseudo-prior, while those conditional on $H_1 : \tau_S < \tau_T$ are from the posterior.

SI text 2. Extended rubber-band algorithm for the MSC-M model. We extend the rubber-band algorithm for changing the species divergence time to accommodate migration events on gene trees. The rubber-band algorithm was developed under the simple MSC model with no gene flow (6) and modifies a species divergence time (τ) by making coordinated changes to coalescent times on the gene trees to avoid conflicts, using an analogy with the movements of points on a rubber band when the rubber band is pulled in one direction or the other. Here we modify the algorithm so that both migration times and coalescent times on the gene trees are changed to avoid conflicts with the proposed species divergence time.

Consider updating the age of the focal node X on the species tree, which has mother node Y and daughter nodes U and V (fig. S1a). We identify bounds (τ_l, τ_u) for τ_X, and the species in which the coalescent times and migration times on gene trees have to be rescaled together with τ_X to avoid conflicts, according to equations A.7 and A.8 of ref. (6). The algorithm proposes a joint change to one species-tree node age and potentially multiple gene-tree node ages and migration times, but keeps the relative order of coalescent and migration events on the gene trees unchanged.

The algorithm identifies the widest bounds $\tau_l < \tau_X < \tau_u$ for which there are no conflicts; wider bounds allow larger changes to τ_X to be proposed. Given the bounds, we identify so-called affected populations, within which gene-tree nodes are affected by the move and have to be rescaled. Note that changes to gene trees cause changes to the sequence likelihood and increase the chance of rejection of the proposed move. Thus we include a population as affected only if it is necessary for avoiding conflicts.

In our algorithm, we may break the duration of a recipient species (i.e., a species that receives immigrants at some loci) into multiple time segments, as the migration rates into that population (i.e., rate M_{sj} into species j in eq. 2) may differ among time segments. For example, in the species tree of figure S1a, species T exists over the time interval (τ_T, τ_S), and this time interval is broken into two segments: (τ_T, τ_X), during which T is receiving migrants from population U, and (τ_X, τ_S), during which T is not a recipient population. In contrast, species U in figure S1a exists over (τ_U, τ_X) and this is just one time segment, during which U is not a recipient population. The algorithm determines whether any migration time on the gene trees falls within each time segment on the species tree defined this way. Breaking one species into multiple time segments in this way facilitates the calculation of the gene-tree density under the MSC-M model (eq. 2) and also allows us to identify fewer affected populations in which the gene-tree nodes have to be rescaled.

We use the ages of the mother node and oldest daughter node of node X to form the initial bounds (τ_l, τ_u). If there are migration events between any of species X, U and V and another species on the species tree, the initial bounds (τ_l, τ_u) may be too wide and may have to be refined. Note that both final bounds (τ_l, τ_u) must coincide with ages of nodes on the species tree. We use the initial bounds to build a list of *linked populations*, which are populations that exchange migrants with any of X, U and V with migration events in either direction on any gene tree during the time interval (τ_l, τ_u), or are linked to another such population. A list of linked populations is constructed for each locus and the lists among loci are assembled. A population is linked to a focal population (X, U, or V) if they are linked at at least one locus. The resulting list is used to determine the bounds for τ_X. We scan the list of linked populations to build two sets of population node ages: the mother set $\mathcal{U}$ and the daughter set $\mathcal{L}$. Then the minimum in $\mathcal{U}$ will be the final upper bound τ_u and the maximum in $\mathcal{L}$ will be the final lower bound τ_l.

Given the bounds (τ_l, τ_u), we identify populations which have gene-tree nodes that are affected by the move and have to be rescaled (6, eqs. A7&A8). These are called the *affected populations*, and gene-tree nodes in affected populations that have to be rescaled are called *affected nodes*. Affected populations are those that fall within the range (τ_l, τ_u) and are a subset of the linked populations. Affected populations are used to identify affected nodes on the gene trees for rescaling. We use locus-specific lists of linked populations to identify affected nodes.

In detail, our extended rubber-band algorithm for proposing a change to the age τ_X of species tree node X involves the following steps:

1. *Initial bounds*. We set the initial τ_l to the age of the older daughter node and τ_u to the age of the mother node. In the example of figure S1a, we have the initial bounds for τ_X to be $(\tau_l, \tau_u) = (\tau_V, \tau_Y)$.

2. *Linked populations and age sets*. Let the branch α-β be a linked population, with α to be the mother node and β the daughter node. If the branch brackets τ_X, with $\tau_\beta < \tau_X < \tau_\alpha$, set $\tau_\alpha \in \mathcal{U}$ and $\tau_\beta \in \mathcal{L}$. If $\tau_\alpha < \tau_X$, set $\tau_\alpha \in \mathcal{L}$. If $\tau_\beta > \tau_X$, set $\tau_\beta \in \mathcal{U}$. Let $\tau_l = \max(\mathcal{L})$ and $\tau_u = \min(\mathcal{U})$. In figure S1a, the linked populations are T and A. Note that D is not a linked population as migration with U are outside the bounds (τ_V, τ_Y). The daughter set is $\mathcal{L} = (\tau_V, \tau_T, \tau_A)$, with τ_V included as the first element. The mother set is $\mathcal{U} = (\tau_Y, \tau_S, \tau_R)$. We thus have $\tau_l = \max(\mathcal{L}) = \tau_T$, and $\tau_u = \min(\mathcal{U}) = \tau_S$.

3. *Affected populations*. We scan the linked populations to remove those that do not exchange migrants (directly or indirectly) with the three focal populations (X, U, V) during (τ_l, τ_u). The remaining linked populations are the affected populations. In the example of figure S1a, the affected populations are (X, U, V, T, A).

We propose an update to the focal τ_X using a sliding window, reflected into the interval (τ_l, τ_u), and jointly propose updates to the node ages and migration times on the gene trees in the affected populations (as in the original rubber-band algorithm). If no migration events link the three focal populations (X, U, V) with any other populations at any locus (as in fig. S1b), the move will be identical to the original rubber-band move of ref. (6). More scenarios are shown in figure S1b–d.

Methods and Materials

Bayesian simulation to validate the MCMC algorithms. *Three species, one migration rate: comparison with G-PhoCS.* We conducted Bayesian simulations to validate our implementation. Each replicate dataset was generated by sampling model parameters from the prior and those sampled parameter values were then used to simulate gene trees and sequence alignments at multiple loci. Each replicate dataset was then analyzed to generate the posterior using the same prior. The expectation is that the posterior averaged over the replicate datasets should match the prior, if both the simulation and the inference programs are implemented correctly (7).

We used two MSC-M models for three species, with either one or eight migration rates (figs. S6a & S7a). The species tree is $((A, B), C)$, with the internal nodes to be R (the root) and S (the A-B ancestor). The first model assumed one migration event from $C \rightarrow S$. The rate M_{CS} was sampled from the gamma prior, $M_{CS} \sim \mathrm{G}(10, 100)$. The five population size parameters were sampled from the gamma prior, $\theta \sim \mathrm{G}(10, 1000)$. The age of the root is sampled from the gamma prior $\tau_R \sim \mathrm{G}(10, 1000)$. Given τ_R, the age of the younger node is uniform, $\tau_S|\tau_R \sim \mathbb{U}(0, \tau_R)$ (8, eq. 2). As $\tau_R \sim \mathrm{G}(\alpha, \beta)$, τ_S has the density

$$p(\tau_S) = \tfrac{\beta}{\alpha-1}\left[1 - G(\tau_S; \alpha - 1, \beta)\right], \qquad [2]$$

where $G(x; \alpha, \beta)$ is the cumulative distribution function (CDF) for the gamma distribution $\mathrm{G}(\alpha, \beta)$ (7, eq. A1). The prior mean of τ_S is $\mathbb{E}(\mathbb{E}(\tau_S|\tau_R)) = \frac{1}{2}\,\mathbb{E}(\tau_R) = \frac{\alpha}{2\beta}$.

We simulated 500 replicate datasets on the species tree of figure S6a. Each dataset consisted of 250 loci, with 4 sequences per species per locus, and 500 sites in the sequence. Specifically, we sampled τ_R, τ_S from the gamma-Dirichlet prior (8, eq. 2), the five θs from the gamma prior, and M_{CS} from the gamma prior. We then generated a control file using those sampled parameters to simulate a replicate dataset using the `simulate` option of BPP version 4.5 (9, 10). For each locus, the program generates a gene tree with coalescent times under the MSC-M model and then "evolves" sequences along the branches of the gene tree under the JC model (11). Sequences at the tips of the gene tree constitute the data at the locus. Gene trees and sequence alignments at different loci in the same replicate dataset are generated using the same set of parameter values while different replicate datasets are generated using different parameter values sampled from the prior.

Each replicate dataset was then analyzed using bpp under the same priors as used in the simulation. We used a burnin of 32,000 MCMC iterations, then took 10^4 samples, sampling every 50 iterations. Analysis of each replicate dataset took ∼ 3 hours using 2 threads. We then merged the MCMC samples across replicate datasets and used kernel density smoothing to estimate the average posterior density.

We included G-PhoCS (1) in the Bayesian simulation under the same MSC-M model. G-PhoCS is based on MCMCcoal, an earlier version of bpp (6, 12), and uses a different parametrization from bpp4. As a result, the two programs use the same likelihood model but different priors. Both programs assign gamma priors on θ. However, G-PhoCS assigns separate gamma priors on the node ages on the species tree, with the joint prior distribution of node ages generated by automatic truncation to ensure that daughter nodes are younger than mother nodes (12). Furthermore, G-PHocs uses the mutation-scaled migration rate, $\varpi_{ij} = m_{ij}/\mu = 4M_{ij}/\theta_j$. We sampled parameter values according to the priors used by G-PhoCS, converted them into parameters used in bpp to simulate the sequence data using bpp, and then analyzed the data using G-PhoCS with the same priors as used in the simulation. Specifically, we sampled five θ parameters from G(10, 1000). We generate species divergence times from $\tau_R \sim$ G(10, 1000) and $\tau_S \sim$ G(10, 2000). If $\tau_S > \tau_R$, we discarded the values and sampled again; this mimics the implicit truncation applied by the program. We sampled the mutation-scaled migration rate ϖ_{CS} from the gamma prior G(10, 0.25) and calculated the population migration rate as $M_{CS} = \theta_S \varpi_{CS}/4$. The sampled parameter values were used to generate a control file to simulate sequence data at $L = 250$ loci using the `simulate` option of bpp. Each dataset was then analyzed using G-PhoCS with the same priors: i.e., $\varpi_{CS} \sim$ G(10, 0.25), $\theta \sim$ G(10, 1000), $\tau_R \sim$ G(10, 1000) and $\tau_S \sim$ G(10, 2000), with the truncation $\tau_R > \tau_S$ applied implicitly by G-PhoCS. We simulated and analyzed 500 replicate datasets. We used a burnin of 10^5 iterations, and took 10^4 samples, sampling every 200 iterations. Analysis of each replicate dataset took ∼10 hours using one thread.

Three species, eight migration rates. The second MSC-M model we used was a saturated model allowing for migration between every pair of contemporary species on the three-species tree, with eight migration rate parameters (fig. S7a). Migration rates involving the ancestral species S (M_{CS}, M_{SC}) are hard to estimate, and in small datasets (with $L = 250$ loci, say), the posterior for those parameters tends to be dominated by the prior, making Bayesian simulation ineffective. Thus we used $L = 1000$ loci, and simulated 500 replicate datasets. The other settings were the same as for the model of one migration rate. We used a burnin of 32,000 iterations, and then took 10^4 samples, sampling every 50 iterations. Running time varied, with the median to be ∼ 30 hours using 2 threads. We did not include G-PhoCS in the test under the saturated model because of the heavy computational load, but analyzed a dataset of 2000 loci generated under the model for comparison with bpp (table S1).

Simulation under a saturated migration model for three species. We conducted simulations under the saturated migration model with eight migration rates, using a fixed set of parameter values (fig. 3a), to examine whether thousands of loci contain enough information to estimate so many migration rate parameters reliably. Each dataset consisted of $L = 250$, 1000, or 4000 loci, with $S = 4$ sequences per species per locus and $N = 500$ sites in the sequence. For each data size (L), we generated 10 replicate datasets, with a total of 30 datasets simulated.

Each replicate dataset was analyzed using bpp under the MSC-M model to estimate the parameters ($\boldsymbol{\tau}$, $\boldsymbol{\theta}$, and $\boldsymbol{M}$). We assigned gamma priors $\tau_R \sim$ G(2, 100) with mean 0.02 for the age of the species-tree root, G(2, 100) with mean 0.02 for θs, and G(2, 10) with mean 0.2 for the migration rates (Ms). The shape parameter $\alpha = 2$ means that the priors are diffuse. Pilot runs were conducted to determine the length of the chain, and then the same setting was used in all runs. Convergence was confirmed by checking the consistency between runs. We used 32,000 iterations as burnin, and took 5×10^5 samples, sampling every 2 iterations.

Running time was ∼12 hours for 250 loci using two threads, and ∼30 hours and ∼120 hours for 1000 and 4000 loci, respectively, using four threads. In addition, we analyzed a large dataset with L =16,000 loci simulated under the model, taking 4×10^5 samples, sampling every iteration. The run took 158 hours using 9 threads on a server (with Intel Xeon Gold 6136 CPUs).

Simulation under an MSC-M model with three species and two migration rates: comparison with IMa3. We compared bpp with IMa3 by analyzing simulated datasets under the MSC-M models of figure 4a–c with a pair of migration rates on a species tree for three species. For each species tree, 10 replicate datasets were generated under the JC model, each consisting of $L = 500$ loci, $S = 4$ sequences per species per locus, and $N = 500$ sites in the sequence.

Each replicate dataset was then analyzed using bpp and IMa3. For bpp, we assigned the gamma prior G(2, 100) with mean 0.02 for both τ_R and θ. Note that given τ_R, τ_S is uniform over $(0, \tau_R)$. The migration rates are assigned the gamma prior, $M \sim$ G(2, 10), with mean 0.2. The prior means may not match the true values but the priors are diffuse. We used a burn-in of 32,000 MCMC iterations, and took 10^4 samples, sampling every 50 iterations. Analysis of each dataset took ∼30 hours using 2 threads.

We used a special version of IMa3, prepared by Professor Jody Hey and available from `https://github.com/jodyhey/ima3/tree/ZY/` (accessed on 25 May 2023), which assumes the JC mutation model (11) instead of HKY (13) and a constant mutation rate for all loci and which can handle larger datasets than the standard version. The following command-line options was used to run the program:

```
mpirun -np 10 ima3 -i mydata.txt -jhE -jh5 -s11 -c 3 -g priors.txt -j2 -b1000 -L4000 -hn20 -ha0.998 -hb 0.4 \
  -o IMa3.out
```

Diffuse priors were used with the prior means matching the true parameter values as much as possible. IMa3 uses mutation rate per generation for the whole locus (whereas in bpp μ is the mutation rate per generation per site) so that divergence time and population size parameters (θ and τ) are multiplied by the sequence length ($N = 500$). We assigned uniform prior $\mathbb{U}(0, 15)$ with mean 7.5 for $N\theta_A$ and $N\theta_S$ (with the true value $0.015 \times 500 = 7.5$), and $\mathbb{U}(0, 25)$ for $N\theta_B$, $N\theta_C$, and $N\theta_R$. We used $\mathbb{U}(0, 20)$ for $N\tau_R$, and $\mathbb{U}(0, 10)$ for $N\tau_S$. Because of the truncation ($\tau_R > \tau_S$), the prior means are $\frac{110}{9} = 12.22$ and $\frac{40}{9} = 4.44$ (cf: true values 10 and 5). IMa3 defines the migration rate under the backwards-in-time coalescent view, scaled by the mutation rate per locus; in the notation here, this is $w'_{ij} = m_{ji}/(N\mu) = 4M_{ji}/(N\theta_i)$. We assigned exponential priors, with the means to be the true values, as follows.

Model	BPP rate	IMA3 rate
Fig. 4a	$M_{AB} = 0.1$	$\varpi'_{BA} = 4 \times 0.1/(500 \times 0.025) = 0.032$
	$M_{BA} = 0.2$	$\varpi'_{AB} = 4 \times 0.2/(500 \times 0.015) = 0.107$
Fig. 4b	$M_{BC} = 0.1$	$\varpi'_{CB} = 4 \times 0.1/(500 \times 0.025) = 0.032$
	$M_{CB} = 0.2$	$\varpi'_{BC} = 4 \times 0.2/(500 \times 0.025) = 0.064$
Fig. 4c	$M_{CS} = 0.1$	$\varpi'_{SC} = 4 \times 0.1/(500 \times 0.015) = 0.053$
	$M_{SC} = 0.2$	$\varpi'_{CS} = 4 \times 0.2/(500 \times 0.025) = 0.064$

We ran IMA3 with 20 chains on 10 cores, using 1000 burnin steps, 4000 genealogies to save, 100 steps between sampling genealogies, 0.998 for heating curve shape parameter, and 0.4 for lower heating value. Analysis of each replicate dataset using 10 threads took ~40 hours.

Simulation under the stepping-stone and island models. We simulated multilocus sequence data under the stepping-stone and island models of figure 5a&b, and analyzed the data using both BPP and MIGRATE (14). Those population-subdivision models are special cases of the MSC-M (or IM) model, with species divergence times at $\tau = \infty$. We thus used very large τs to simulate data under the MSC-M model (fig. 5a&b), to ensure that all sequences at the locus have coalesced before reaching species divergences when we trace the genealogy of sequences at each locus backwards in time.

Each replicate dataset consists of $L = 250$, 1000, or 4000 loci, with $S = 4$ sequences per species per locus and $N = 500$ sites in the sequence. The number of replicates is 10.

Each replicate dataset was analyzed using BPP under the MSC-M model to estimate the parameters: τs, θs, and Ms. We use gamma priors $\theta \sim$ G(2, 1000) for the population sizes and $\tau_R \sim$ G(2, 20) for the age of the root in the species tree. The migration rates are assigned the gamma prior $M \sim$ G(2, 2/0.15). We used 32,000 MCMC iterations as burnin, and took 2×10^5 samples, sampling every 2 iterations. Running time using 8 threads was 33, 142, and 354 hours at 250, 1000, and 4000 loci for the stepping-stone model, and 8, 47, and 235 hours for the island model.

The small datasets of $L = 250$ loci were also analyzed using MIGRATE 5.0.4 (14) under the stepping-stone and island models (fig. 5e&f). Note that both programs estimate the migration rates ($\boldsymbol{M}$) and the population sizes ($\boldsymbol{\theta}$) for extant populations but BPP in addition estimates the species divergence times ($\boldsymbol{\tau}$) and ancestral population sizes. MIGRATE uses the mutation-scaled migration rate, defined as $\varpi_{ij} = m_{ij}/\mu = 4M_{ij}/\theta_j$. We used the gamma prior $w \sim$ G(2, 2/300), constrained to be in the range $(0, 3000)$. The population sizes were assigned the same gamma prior as in BPP, G(2, 1000), but constrained in the range $(0, 0.05)$. Those bounds are loose and had virtually no effects.

Analysis of the *Anopheles* mosquito genomic data. We analyzed genomic sequence data from six species of African mosquitoes in the *Anopheles gambiae* species complex. Coding and noncoding sequence alignments were compiled from the genomic data of ref. (15) in ref. (16). There are 12 sequences per locus, with two sequences per species. The species tree of figure 6b with two introgression events was built in refs. (10, 16). We replaced introgression by migration to form an MSC-M model (fig. 6a).

We analyzed blocks of 100 loci, as in ref. (16), and then combined loci for each of the eight chromosomal arms/regions: 2L1, 2La (the inversion region on 2L), 2L2, 2R, 3L1, 3La (the inversion region on 3L), 3L2, and 3R. The X chromosome was not used. The priors were $\tau_o \sim$ G(3, 30) with mean 0.1 for the age of the root, $\theta \sim$ G(3, 150) with mean 0.02, and $M \sim$ G(2, 10) with mean 0.2. We used a burn-in of 10^5 iterations, and took 5×10^5 samples, sampling every 2 iterations.

For comparison, we re-analyzed the same data using the MSC-I model implemented in BPP. In the analysis of ref. (10) under the MSC-I model, inverse gamma priors were used for τ and θ and two separate population size parameters for the same branch before and after introgression on the species tree (e.g., θ_b and θ_f in fig. 6b) were assumed. Here we used gamma priors and assumed the same θ before and after the introgression event. We assigned gamma priors $\tau_0 \sim$G(3, 150) and $\theta \sim$ G(3, 30), while the introgression probability was assigned the $\mathbb{U}(0, 1)$ prior.

We then analyzed all loci for each chromosomal arm as one large dataset. We ran each analysis 10 times, to assess the mixing issues.

Each analysis of the 100-loci block took at most two hours. The analysis of the 16 large datasets for the chromosomal arms (table 1) using from 4 to 9 threads on various servers took from 34 to 581 hours (table S3).

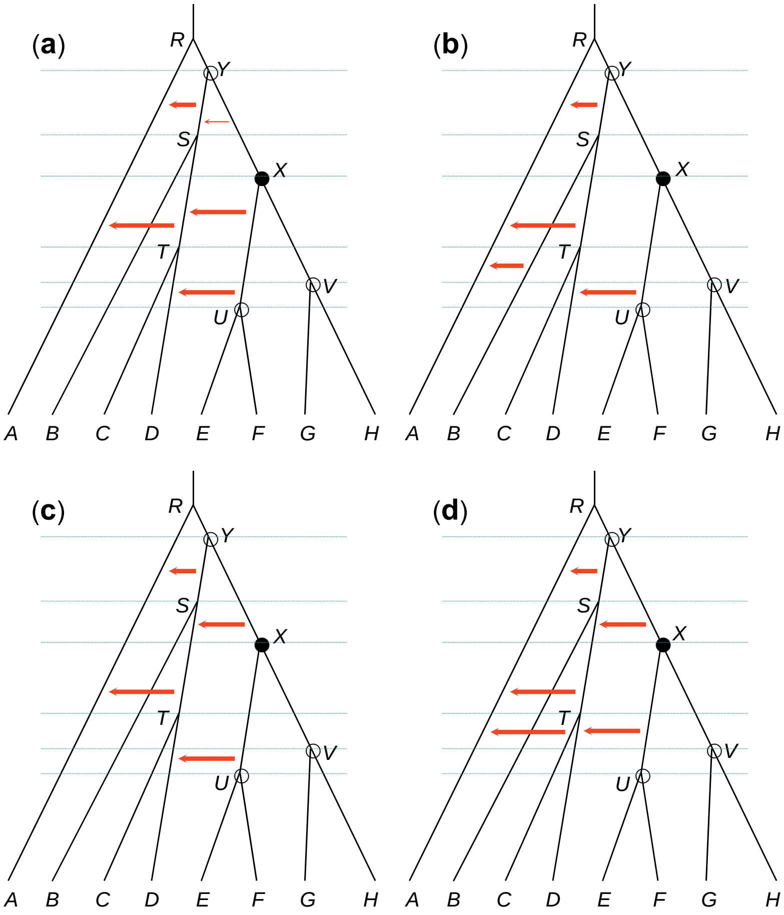

Fig. S1. Four MSC-M models used to illustrate the determination of the bounds (τ_l, τ_u) when species divergence time τ_X is updated in the extended rubber-band algorithm. Red arrows represent migration in the model with rate $M > 0$. Thin red arrows (e.g., the one from X to S in **a**) indicate that no migration event occurs at any locus (gene tree) even if such migration is allowed in the model, while thick arrows indicate presence of migration events at one or more loci. Thick arrows or migrant events are used to identify bounds on τ_X in the algorithm. (**a**) The initial bounds are $(\tau_l, \tau_u) = (\tau_V, \tau_Y)$, the linked populations are (ST, RA), and the sets are $\mathcal{L} = (\tau_V, \tau_T, \tau_A)$, $\mathcal{U} = (\tau_Y, \tau_S, \tau_R)$. The new bounds are $\tau_l = \max(\mathcal{L}) = \tau_T$, $\tau_u = \min(\mathcal{U}) = \tau_S$. The final bounds are (τ_T, τ_S). The affected populations are (X, U, V, T, A). Note that the upper bound τ_u is τ_S rather than τ_Y as using τ_Y as the upper bound might lead to τ_X and the time of migration from populations $XU \to ST$ exceeding τ_S. Also gene-tree node ages in population T older or younger than τ_X are rescaled according to eqs. A7&A8 in ref. (6), respectively, as are those in population A. Even if there are no migration events in T in (τ_X, τ_S), node ages in T in that time interval are changed as well. (**b**) The initial bounds are (τ_V, τ_Y), there are no linked populations, and the final bounds are (τ_V, τ_Y). The extended algorithm will then be equivalent to the original algorithm of ref. (6). (**c**) The initial bounds are (τ_V, τ_Y), the linked populations are (ST, RA), and the sets are $\mathcal{L} = (\tau_V, \tau_T, \tau_A)$, $\mathcal{U} = (\tau_Y, \tau_S, \tau_R)$. The new bounds are (τ_T, τ_S). The affected populations are (X, U, V, T, A). (**d**) The initial bounds are (τ_V, τ_Y), the linked populations are (TD, ST, RA), and the sets are $\mathcal{L} = (\tau_V, \tau_T, \tau_T, \tau_A)$, $\mathcal{U} = (\tau_Y, \tau_S, \tau_R)$. The new bounds are (τ_T, τ_S). The affected populations are (X, U, V, T, A).

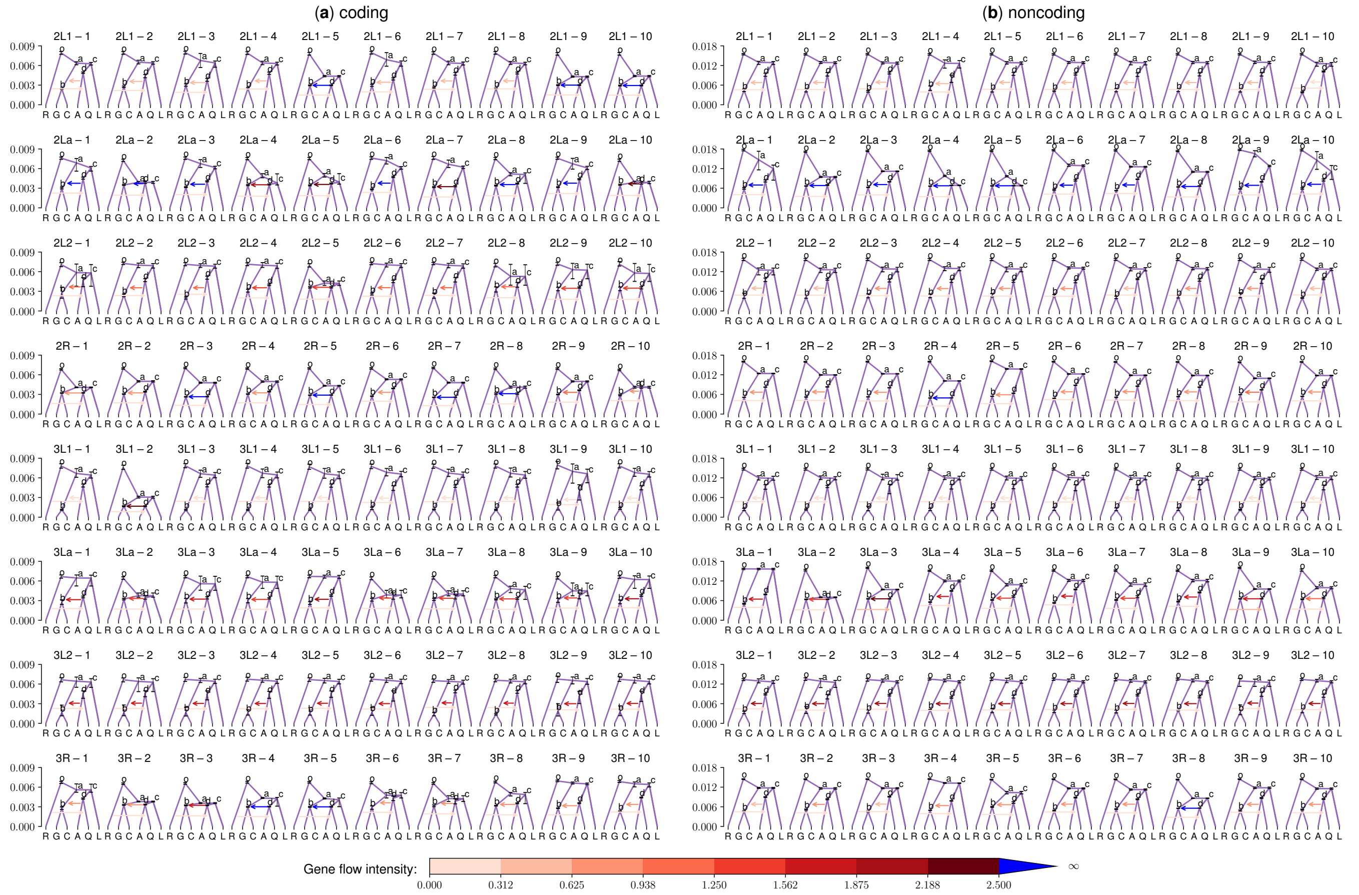

Fig. S2. Rejection algorithm. Estimated species divergence times and migration rates under the MSC-M model from 10 replicate BPP runs of the rejection algorithm in the analysis of the (**a**) coding and (**b**) noncoding datasets from the eight chromosomal arms (fig. S12). The y-axis (and vertical bars for the CIs) indicates the divergence times (τ) while the colour intensity of the horizontal arrows indicates the migration rate (M). The 10 runs for each analysis are in the columns. Trace and box plots for the first dataset (2L1 coding) are shown in figure S4.

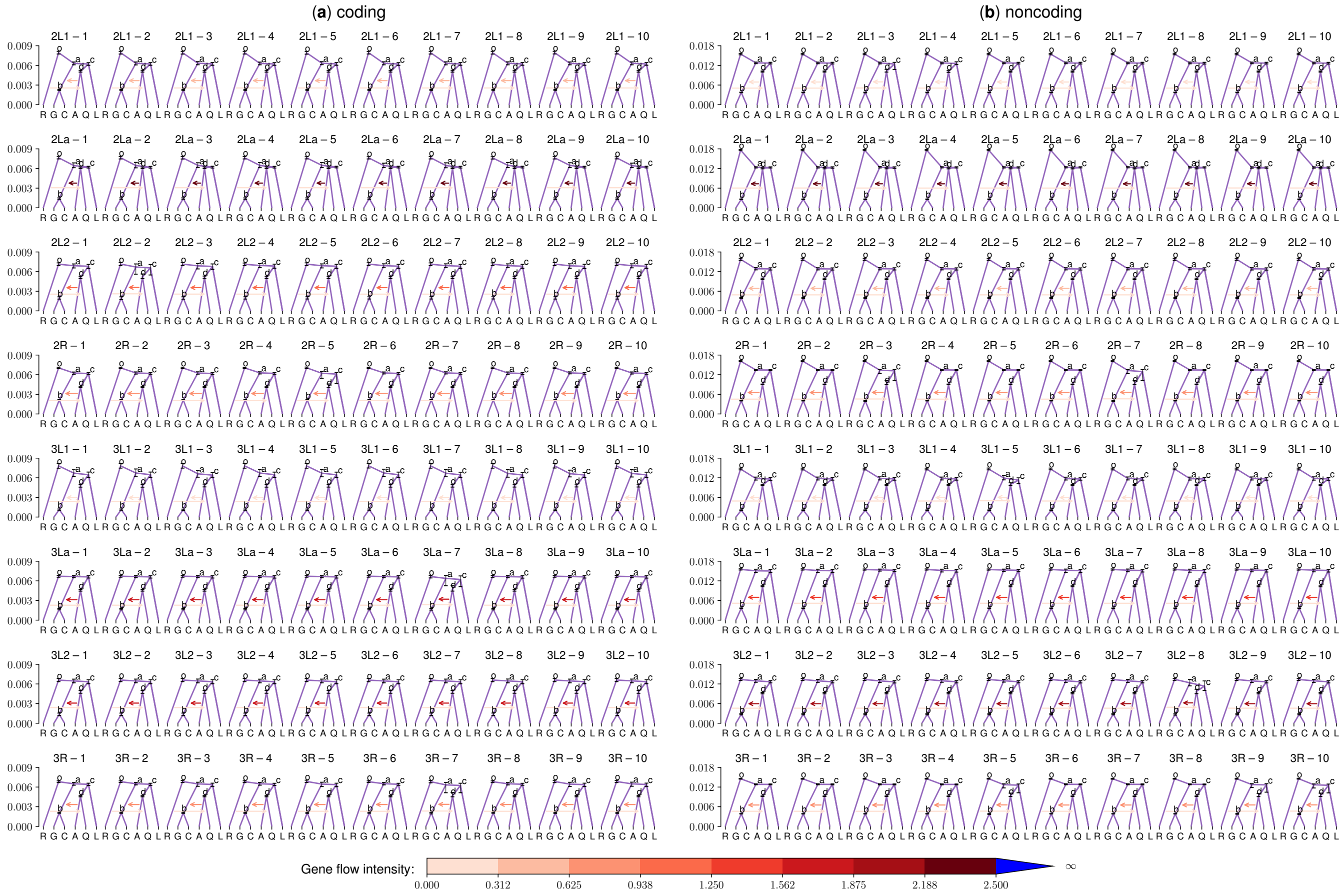

Fig. S3. Extended rubber-band algorithm. Estimated species divergence times and migration rates using the extended rubber-band algorithm for the same data of figure S2. Trace and box plots for the first dataset (2L1 coding) are shown in figure S5.

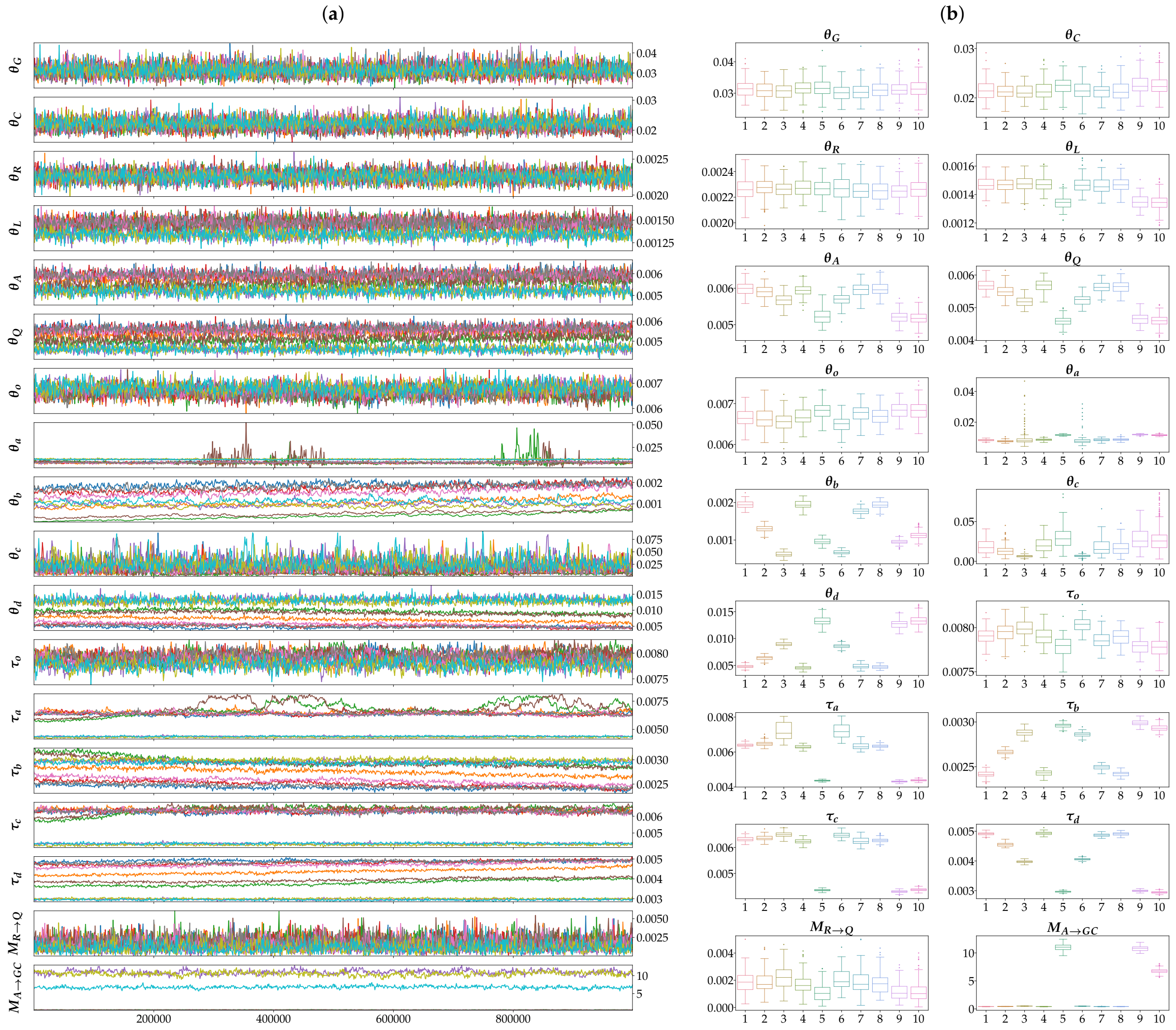

Fig. S4. Rejection algorithm. (**a**) Trace and (**b**) box plots for parameters in the MSC-M model in 10 BPP runs using the rejection algorithm for updating τ in the analysis of the 2223 coding loci from chromosomal arm 2L1 (fig. 6a). The large differences among runs suggest mixing issues.

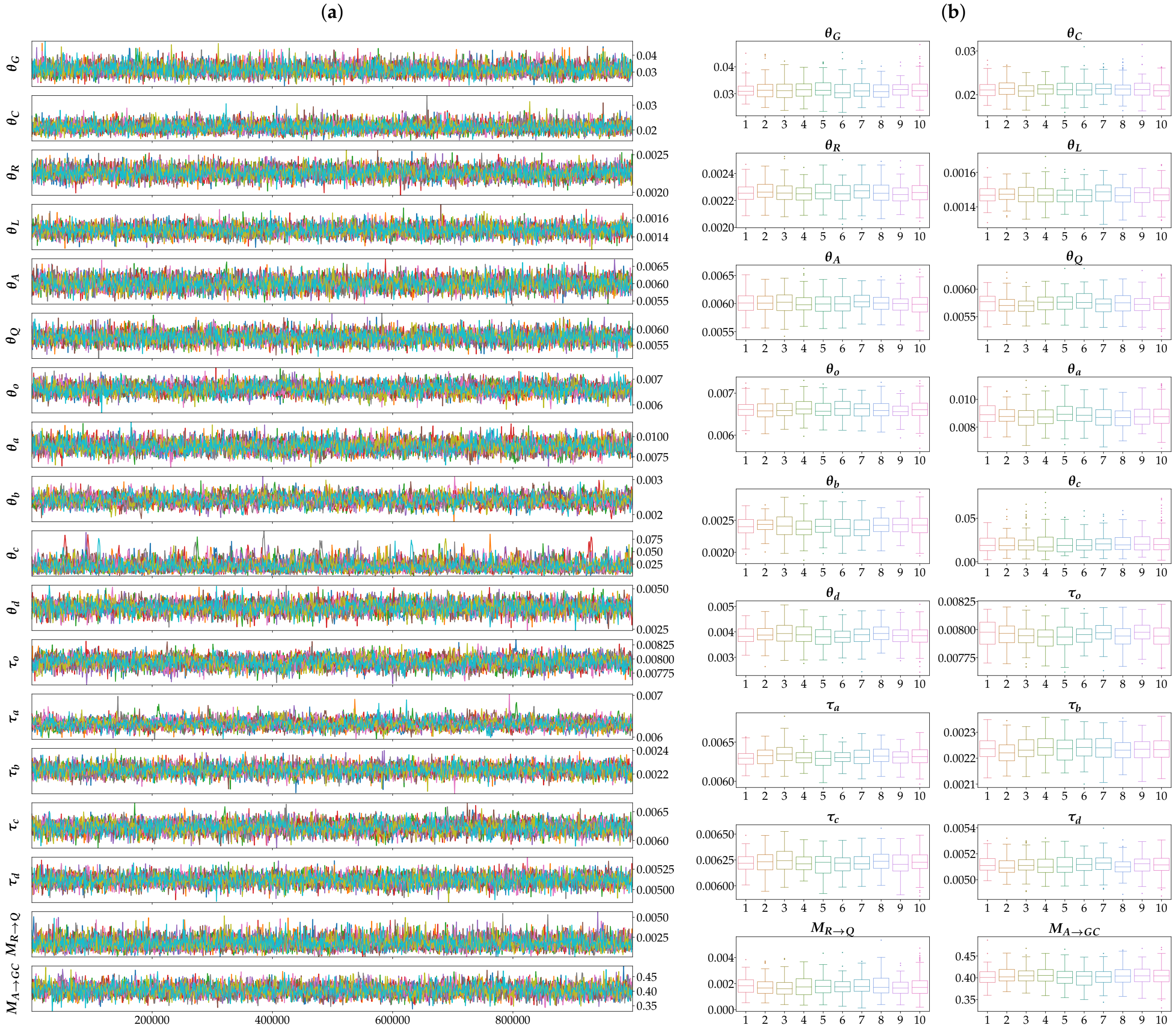

Fig. S5. Extended rubber-band algorithm. (**a**) Trace and (**b**) box plots for parameters in the MSC-M model in 10 BPP runs using the extended rubber-band algorithm in the analysis of the same data as in figure S4.

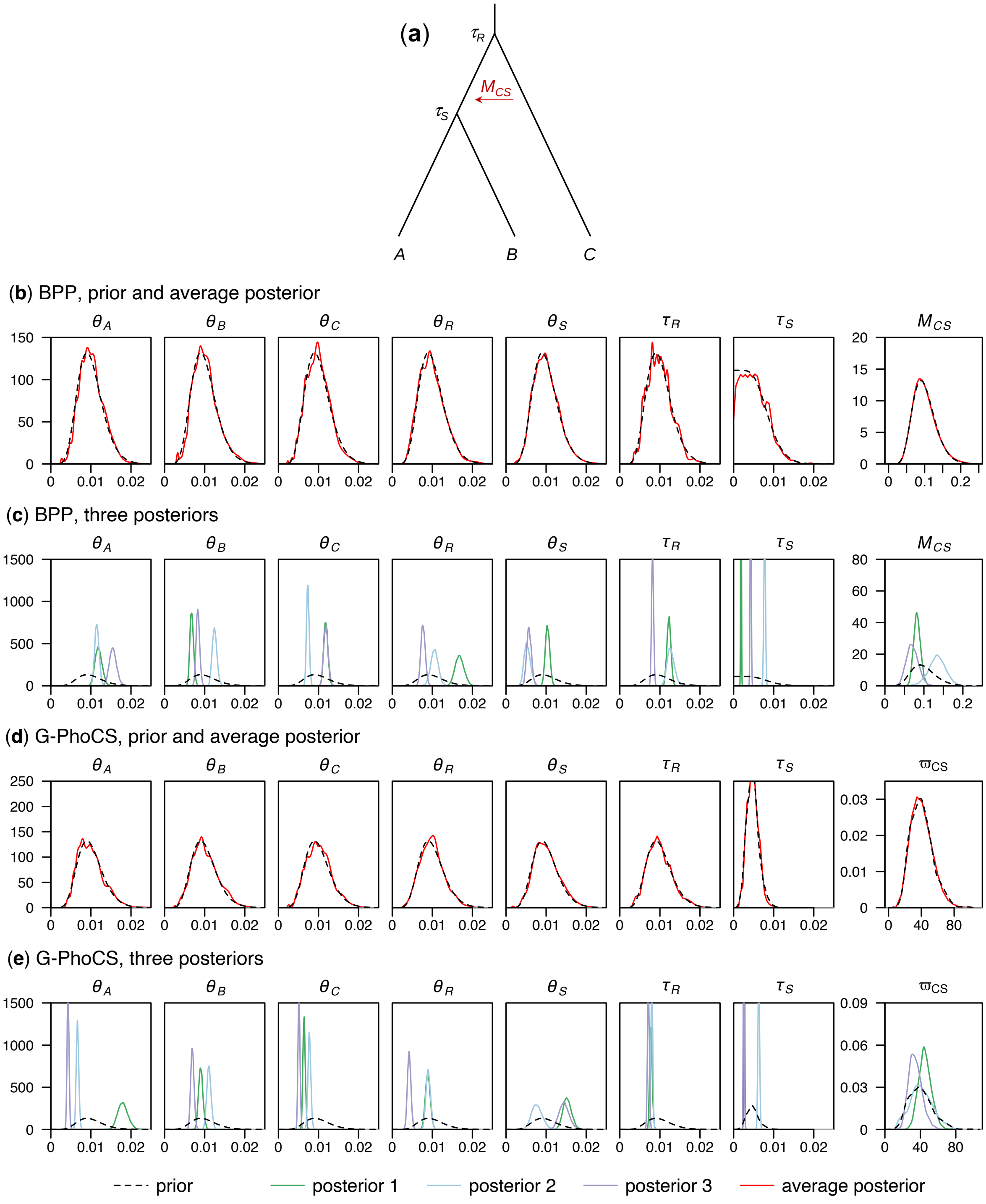

Fig. S6. (**a**) MSC-M model for three species with $C \to S$ migration, used in Bayesian simulation to validate BPP and G-PhoCS. (**b**) Prior (black dashed line) and average posterior (red solid line) distributions of eight parameters in the model in BPP analysis of the data. The prior for τ_S is given by eq. 2. (**c**) Posterior distributions from three replicate datasets from BPP. Averaging over posterior distributions over replicate datasets recovers the prior in (**b**). (**d**) Prior (black dashed line) and average posterior (red solid line) for G-PhoCS. The priors for τ_R and τ_S were generated by simulating 10^6 pairs of gamma variables and applying truncation so that $\tau_R > \tau_S$. The migration rate is defined as $\varpi_{CS} = 4M_{CS}/\theta_S$. (**e**) Three posterior distributions for three datasets by G-PhoCS.

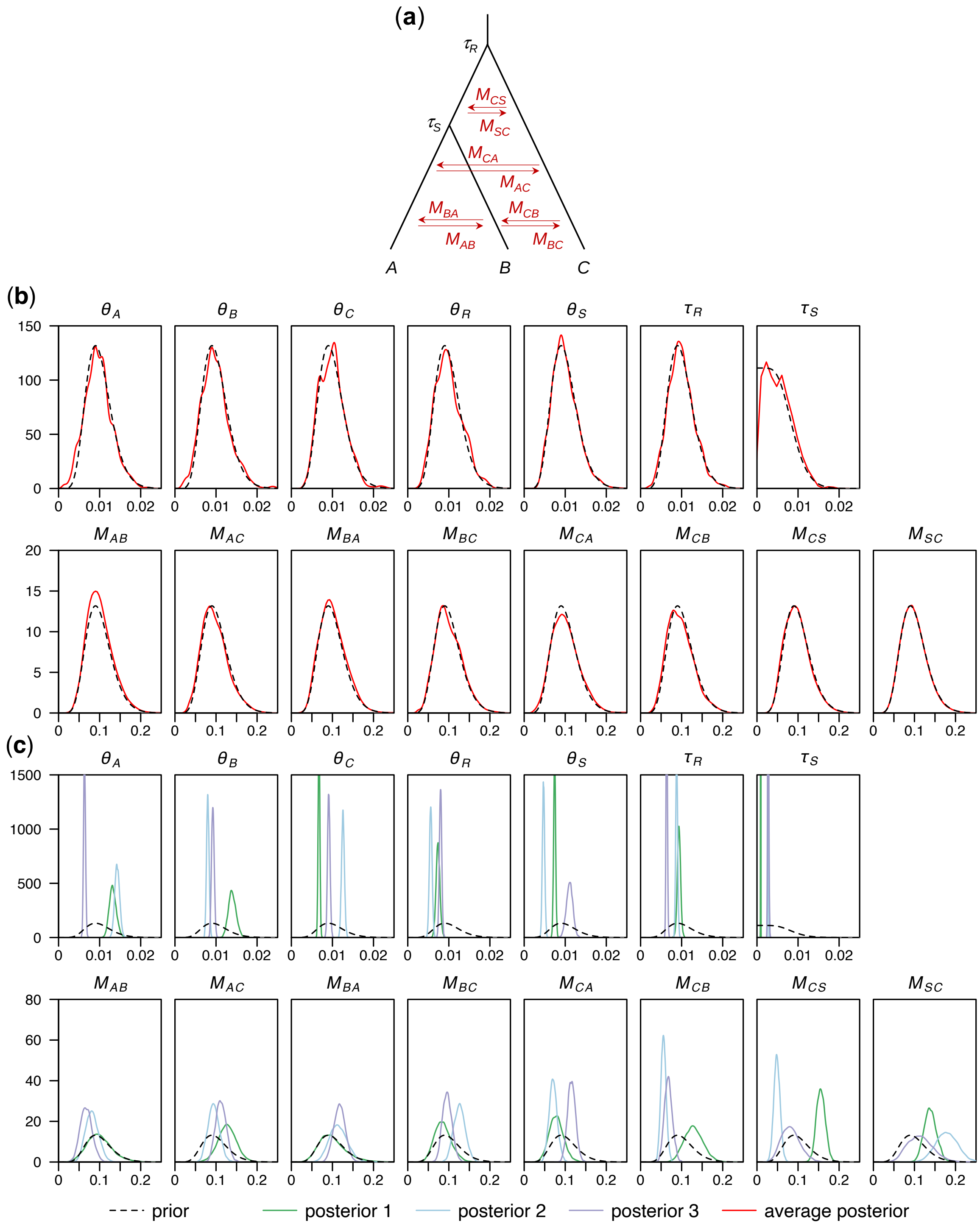

Fig. S7. (**a**) Saturated migration model for three species with eight migration rates, used in Bayesian simulation to validate BPP. (**b**) Prior (black dashed line) and average posterior (red solid line) for 15 parameters in BPP analysis of the data. (**c**) Posterior distributions from three replicate datasets.

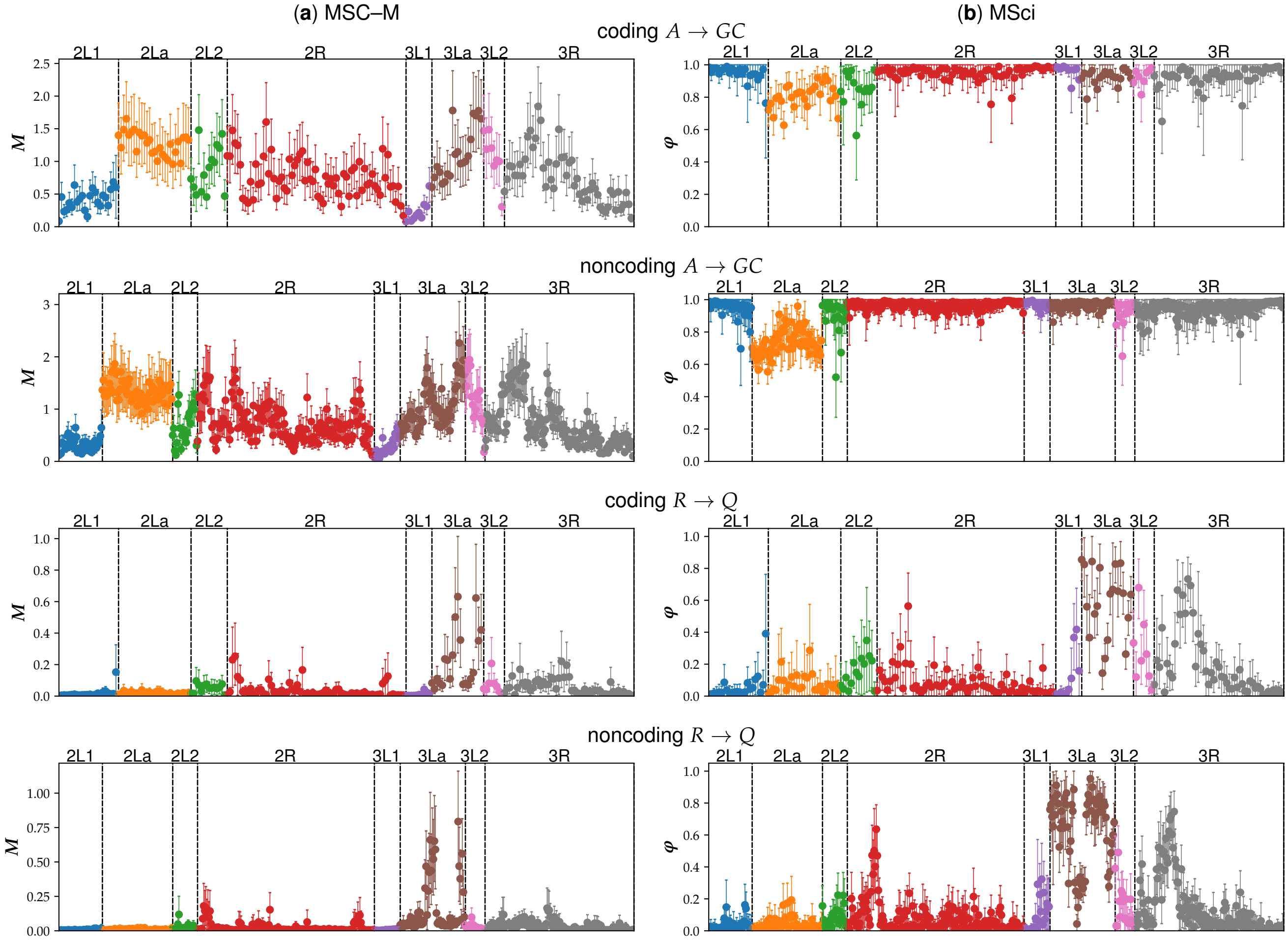

Fig. S8. (**a**) **MSC-M.** Posterior means and 95% HPD CIs of migration rates, $M_{A \to GC}$ and $M_{R \to Q}$ (fig. 6a), obtained from BPP analysis of the 100-loci blocks. (**b**) **MSC-I.** Introgression probabilities ($\varphi_{A \to GC}$, $\varphi_{R \to Q}$ in the MSC-I model, fig. 6b) under the MSC-I model. The MSC-I results are very similar to those of ref. (10, fig. 6), where inverse gamma priors were used for τ and θ. Here we used gamma priors, and assumed the same population size before and after each introgression event ($\theta_R = \theta_g$, $\theta_b = \theta_f$, etc.; fig. 6b).

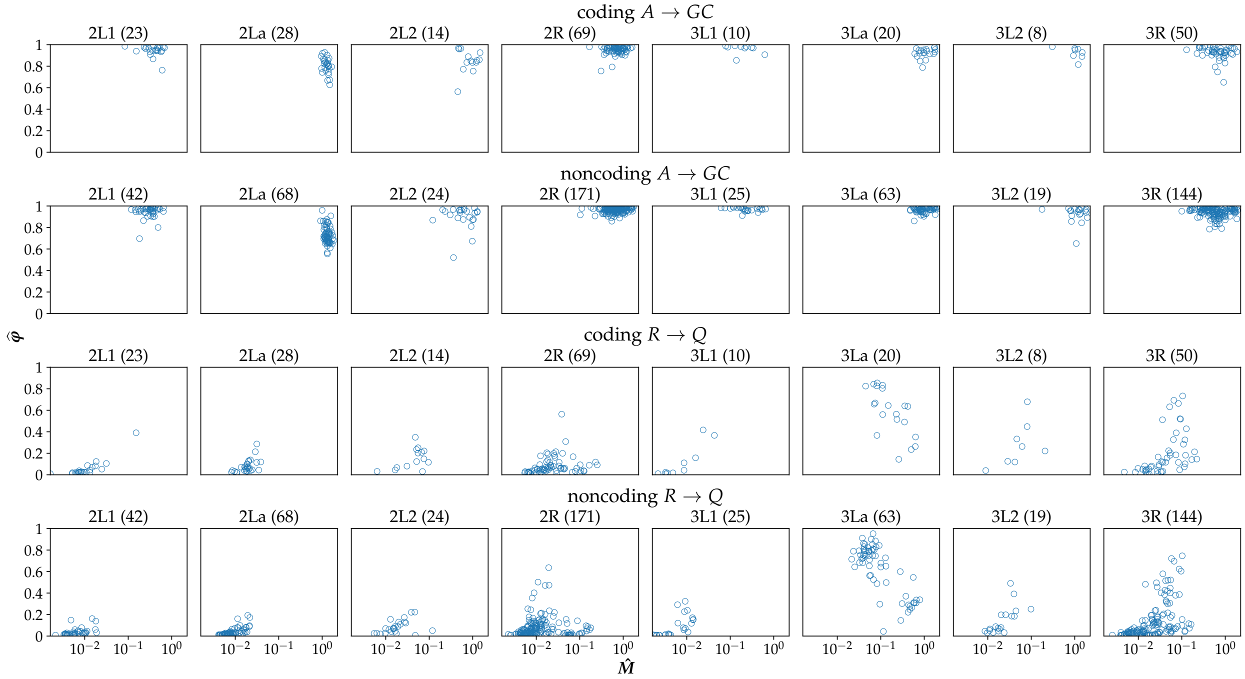

Fig. S9. Posterior mean introgression probability ($\hat{\varphi}$) under the MSC-I model plotted against posterior mean migration rate ($\hat{M}$) under the MSC-M model in BPP analysis of the 100-loci blocks. Note that $\hat{M}$ is on logarithmic scale. The estimates are shown along the chromosomes in figure S8a.

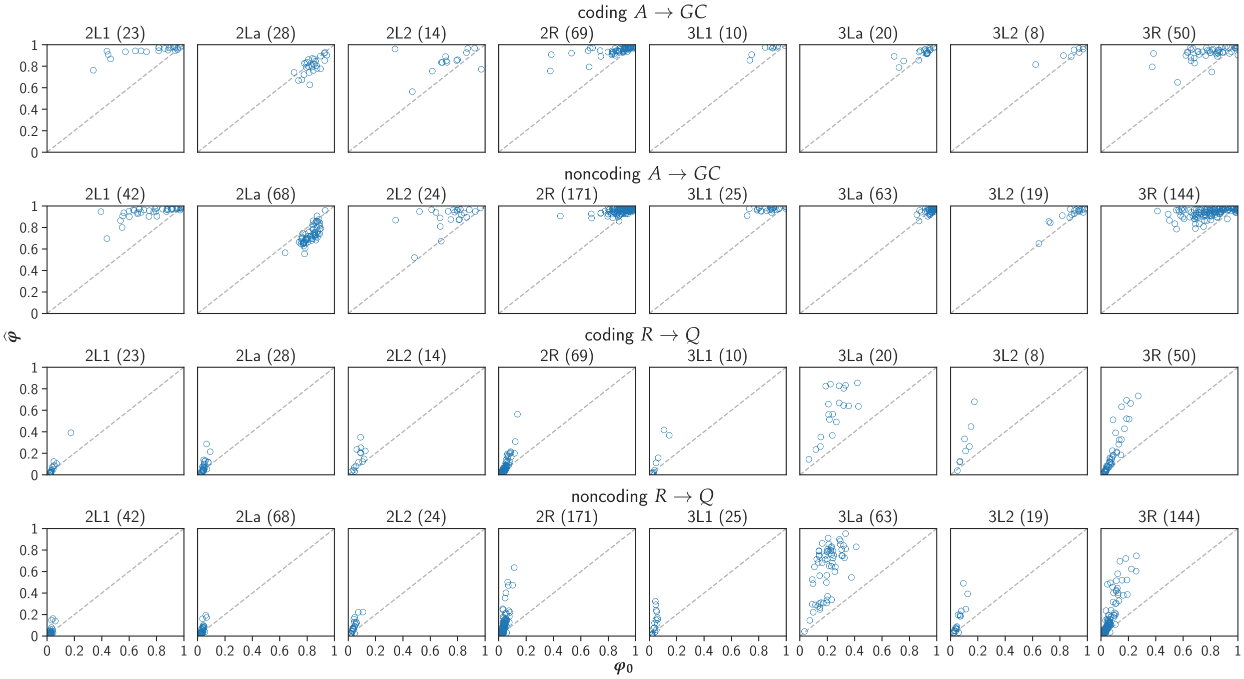

Fig. S10. Posterior mean introgression probability under the MSC-I model ($\hat{\varphi}$) plotted against the expected introgression probability (φ_0) calculated using parameter estimates under the MSC-M model in BPP analysis of the 100-loci blocks: $\varphi_{0,A\to GC} = 1 - \exp\left\{-\frac{4M_{A\to GC}}{\theta_b}(\tau_d - \tau_b)\right\}$ and $\varphi_{0,R\to Q} = 1 - \exp\left\{-\frac{4M_{R\to Q}}{\theta_Q}\tau_d\right\}$ (ref. (17), eq. 10).

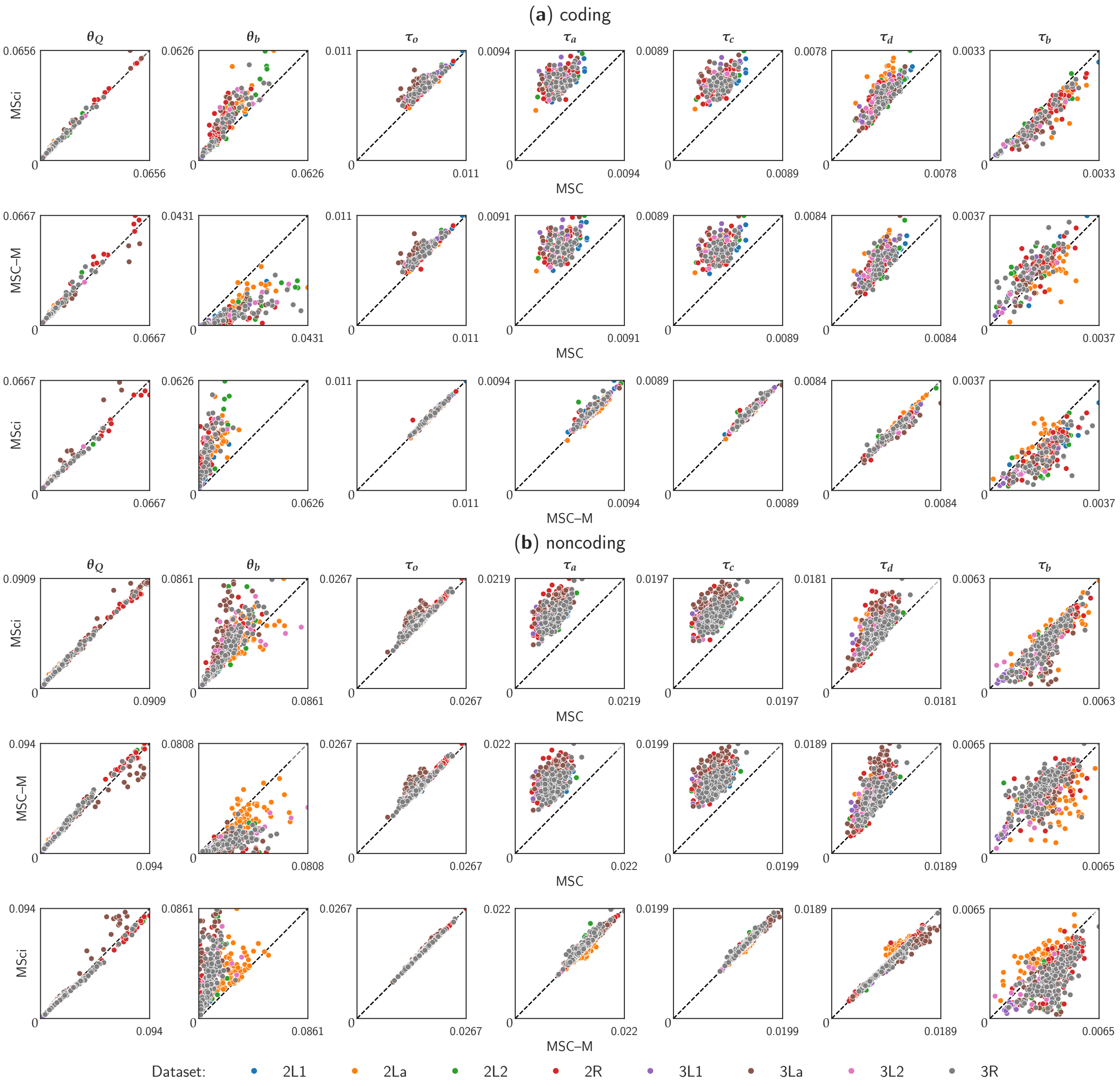

Fig. S11. Posterior means of parameters under three models from the 100-loci blocks of the (**a**) coding and (**b**) noncoding data from *Anopheles*: the MSC model with no gene flow, the migration (MSC-M) model, and the introgression (MSC-I) model (fig. 6a&b). Blocks from different chromosomal regions are represented by different colours, as in figure S8.

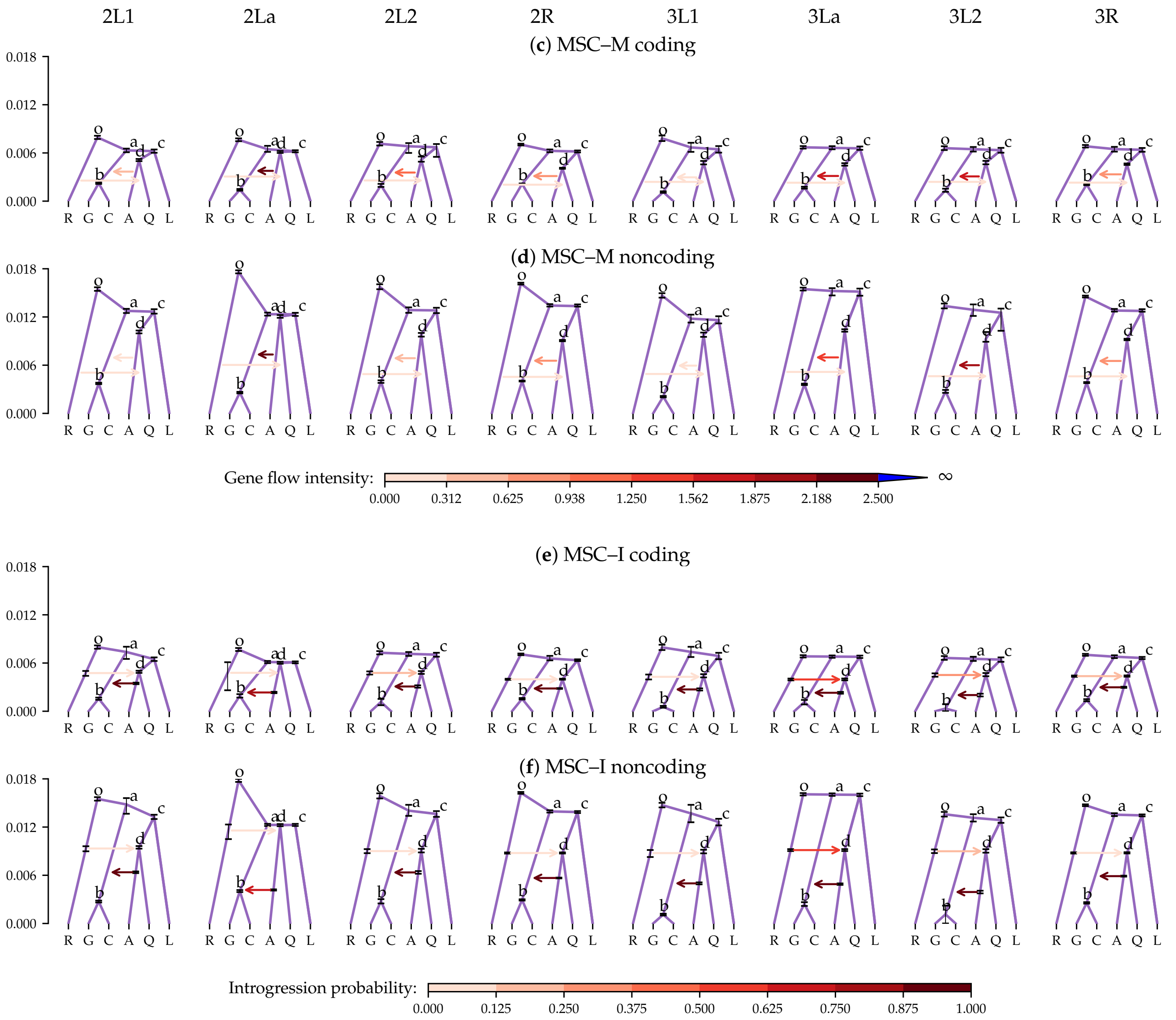

Fig. S12. (**a**&**b**) Species trees showing estimated divergence times and migration rates (indicated by the intensity of the colour) under the MSC-M model in BPP analysis of the (**a**) coding and (**b**) noncoding loci from the chromosomal arms. (**c**&**d**) Estimates under the MSC-I model. See table 1 for estimates of all parameters.

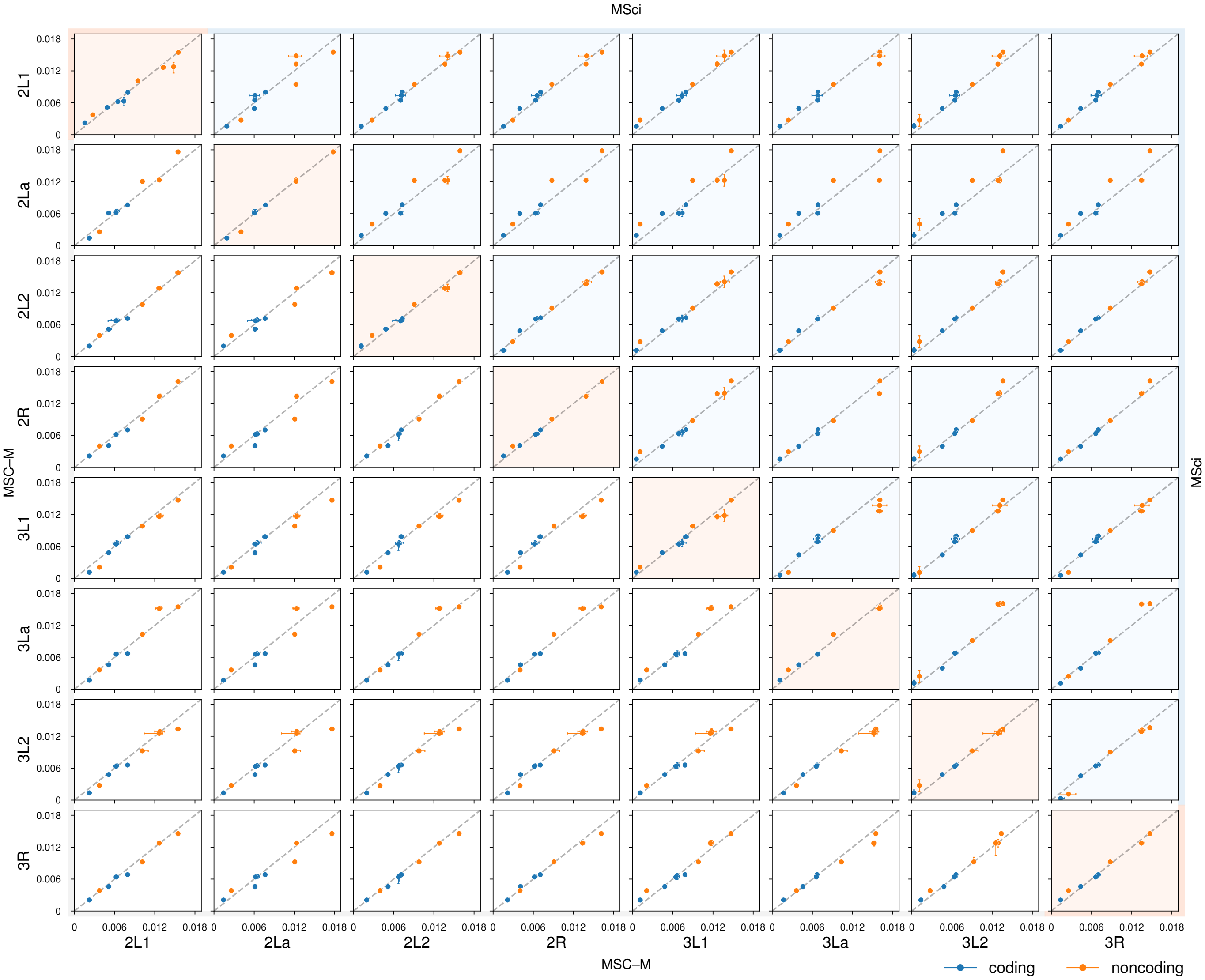

Fig. S13. Posterior means and 95% HPD CIs of divergence times (τ) under the MSC-M and MSC-I models in analysis of the coding (blue) and noncoding (orange) datasets for the chromosomal arms from *Anopheles*. The lower triangle has MSC-M against MSC-M estimates, the diagonal (with red frame) has MSC-M (y-axis) against MSC-I (x-axis), while the upper triangle has MSC-I against MSC-I. The five points for each dataset correspond to (from large to small): τ_o, τ_a, τ_c, τ_d, and τ_b (fig. 6a).

Table S1. Posterior means and 95% HPD CIs (in parentheses) for parameters obtained in BPP and G-PhoCS analyses of two datasets (of L = 2000 or 16,000 loci) simulated under the saturated migration model of figure 3a.

				L = 2000, G-PhoCS		
	Truth	L = 16000, BPP	L = 2000, BPP	Truth	run 1	run 2
θ_A	0.015	0.0148 (0.0145, 0.0151)	0.0153 (0.0145, 0.0162)	0.015	0.0151 (0.0142, 0.0159)	0.0151 (0.0143, 0.0160)
θ_B	0.025	0.0254 (0.0249, 0.0258)	0.0260 (0.0247, 0.0273)	0.025	0.0264 (0.0250, 0.0277)	0.0265 (0.0252, 0.0278)
θ_C	0.025	0.0247 (0.0243, 0.0252)	0.0238 (0.0227, 0.0250)	0.025	0.0239 (0.0227, 0.0251)	0.0237 (0.0226, 0.0250)
θ_R	0.025	0.0250 (0.0246, 0.0254)	0.0244 (0.0232, 0.0255)	0.025	0.0257 (0.0245, 0.0269)	0.0259 (0.0247, 0.0270)
θ_S	0.015	0.0151 (0.0142, 0.0160)	0.0166 (0.0142, 0.0189)	0.015	0.0200 (0.0175, 0.0226)	0.0206 (0.0180, 0.0231)
τ_R	0.020	0.0203 (0.0200, 0.0207)	0.0204 (0.0196, 0.0212)	0.020	0.0183 (0.0175, 0.0190)	0.0181 (0.0174, 0.0188)
τ_S	0.010	0.0102 (0.0099, 0.0104)	0.0101 (0.0096, 0.0107)	0.010	0.0092 (0.0086, 0.0097)	0.0091 (0.0085, 0.0096)
M_{AB}	0.12	0.1193 (0.1067, 0.1317)	0.1299 (0.0949, 0.1661)	$\varpi_{AB} = 19$	12.5 (7.7, 17.3)	12.5 (7.6, 17.2)
M_{AC}	0.13	0.1341 (0.1238, 0.1451)	0.1189 (0.0883, 0.1500)	$\varpi_{AC} = 21$	16.0 (11.2, 20.7)	16.6 (12.2, 21.2)
M_{BA}	0.21	0.2214 (0.2115, 0.2312)	0.2297 (0.2008, 0.2589)	$\varpi_{BA} = 56$	58.8 (50.0, 68.2)	58.9 (50.1, 67.8)
M_{BC}	0.23	0.2361 (0.2234, 0.2495)	0.2399 (0.2050, 0.2741)	$\varpi_{BC} = 37$	38.6 (33.3, 44.0)	39.1 (33.8, 44.7)
M_{CA}	0.31	0.3011 (0.2904, 0.3117)	0.2851 (0.2554, 0.3149)	$\varpi_{CA} = 83$	75.2 (67.2, 83.2)	74.5 (67.1, 82.3)
M_{CB}	0.32	0.3227 (0.3080, 0.3377)	0.2990 (0.2632, 0.3358)	$\varpi_{CB} = 51$	45.9 (40.4, 51.5)	45.5 (40.1, 51.1)
M_{CS}	0.20	0.2075 (0.1728, 0.2386)	0.1998 (0.1112, 0.2834)	$\varpi_{CS} = 53$	8.2 (0.1, 17.4)	7.4 (0.5, 16.5)
M_{SC}	0.10	0.1381 (0.0868, 0.1816)	0.1220 (0.0219, 0.2291)	$\varpi_{SC} = 16$	3.9 (0.1, 9.3)	3.9 (0.1, 9.6)

Note.— The 16,000-loci dataset is analyzed using BPP only, while the 2000-loci dataset is analyzed using both BPP and G-PhoCS. G-PhoCS uses the same definitions of τs and θs, but the mutation-scaled migration rate: $\varpi_{ij} = m_{ij}/\mu = 4M_{ij}/\theta_j$. In the G-PhoCS analysis, the priors are chosen to be close to those used by BPP: $\tau_R \sim G(2, 100)$, $\tau_S \sim G(2, 200)$, $\theta \sim G(2, 100)$ for all populations, and $\varpi \sim G(2, 0.05)$ with mean 40 for all ϖ_{ij}.

Table S2. Posterior means, 95% HPD CI width, and CI coverage for parameters in the stepping-stone and island models (fig. 5a&b) averaged over 10 replicate datasets

		L = 250 loci			L = 1,000 loci			L = 4,000 loci		
	Truth	mean	CI width	coverage	mean	CI width	coverage	mean	CI width	coverage
**Stepping-stone model (fig. 5a)**										
θ_A	0.002	0.00202	0.000527	0.9	0.00195	0.000255	0.7	0.00205	0.000128	0.6
θ_B	0.002	0.00201	0.000564	1.0	0.00200	0.000294	0.7	0.00196	0.000139	0.4
θ_C	0.002	0.00200	0.000579	0.9	0.00205	0.000292	0.7	0.00202	0.000145	0.6
θ_D	0.002	0.00200	0.000524	0.9	0.00203	0.000262	0.7	0.00198	0.000130	0.5
θ_R	0.002	0.00200	0.00473	1.0	0.00198	0.00468	1.0	0.00195	0.00467	1.0
θ_T	0.002	0.00199	0.00471	1.0	0.00198	0.00468	1.0	0.00190	0.00451	1.0
θ_S	0.002	0.00200	0.00473	1.0	0.00199	0.00471	1.0	0.00204	0.00478	1.0
τ_R	0.100	0.1128	0.10242	0.8	0.1168	0.09600	0.7	0.1083	0.07441	0.8
τ_T	0.098	0.0837	0.03146	0.3	0.0884	0.01637	0.2	0.0865	0.01031	0.3
τ_S	0.096	0.0630	0.02992	0.3	0.0619	0.01455	0.0	0.0666	0.00949	0.0
M_{AB}	0.15	0.1429	0.0572	1.0	0.1469	0.0289	0.8	0.1567	0.0149	0.4
M_{BA}	0.15	0.1490	0.0561	0.9	0.1546	0.0285	0.7	0.1442	0.0132	0.5
M_{BC}	0.15	0.1587	0.0564	0.9	0.1499	0.0274	0.8	0.1493	0.0136	0.6
M_{CB}	0.15	0.1487	0.0531	0.8	0.1535	0.0280	0.7	0.1542	0.0140	0.4
M_{CD}	0.15	0.1498	0.0555	1.0	0.1486	0.0276	0.8	0.1560	0.0146	0.7
M_{DC}	0.15	0.1567	0.0614	0.8	0.1516	0.0292	0.5	0.1468	0.0142	0.7
**Island model (fig. 5b)**										
θ_A	0.020	0.01934	0.002373	0.7	0.01990	0.001226	0.9	0.01999	0.000615	1.0
θ_B	0.002	0.00207	0.000579	0.9	0.00203	0.000282	0.8	0.00200	0.000141	0.8
θ_C	0.002	0.00206	0.000572	1.0	0.00203	0.000288	0.9	0.00201	0.000141	0.9
θ_D	0.002	0.00205	0.000567	1.0	0.00198	0.000278	0.9	0.00198	0.000140	1.0
θ_R	0.002	0.00200	0.004713	1.0	0.00200	0.004727	1.0	0.00200	0.004725	1.0
θ_T	0.002	0.00200	0.004718	1.0	0.00200	0.004717	1.0	0.00201	0.004750	1.0
θ_S	0.002	0.00205	0.004836	1.0	0.00206	0.004870	1.0	0.00250	0.006704	1.0
τ_R	0.100	0.1592	0.2219	1.0	0.1687	0.2111	0.8	0.1686	0.2031	0.8
τ_T	0.098	0.1205	0.1596	1.0	0.1294	0.1454	1.0	0.1293	0.1343	1.0
τ_S	0.096	0.0819	0.0819	0.8	0.0910	0.0595	0.7	0.0900	0.0319	0.5
M_{AB}	0.15	0.1544	0.0480	1.0	0.1503	0.0235	0.9	0.1492	0.0118	1.0
M_{AC}	0.15	0.1521	0.0475	0.9	0.1550	0.0244	0.9	0.1502	0.0119	1.0
M_{AD}	0.15	0.1521	0.0473	1.0	0.1474	0.0233	0.8	0.1479	0.0117	0.9

Note.— The 95%HPD CIs from the replicate datasets are plotted in figure 5c&d.

Table S3 Running times on different servers in analysis of the Anopheles genomic datasets

2L1 (2223)			2La (2776)			2L2 (1362)			2R (6849)			2L1+2 (3585)			3L1 (983)			3La (1998)			3L2 (764)			3R (4977)			3L1+2 (1747)		
Machine	Cores	Time	Machine	Cores	Time	Machine	Cores	Time	Machine	Cores	Time	Machine	Cores	Time	Machine	Cores	Time	Machine	Cores	Time	Machine	Cores	Time	Machine	Cores	Time	Machine	Cores	Time
okapi	4	78:02:39	indri	6	77:19:43	llama	4	43:09:29	okapi	4	248:42:40	potto	6	91:58:31	okapi	4	35:26:38	indri	6	55:45:29	okapi	4	30:45:36	potto	6	138:08:31	indri	6	46:47:25
okapi	4	77:43:27	indri	6	75:12:42	llama	4	43:01:01	okapi	4	245:54:26	potto	6	91:13:55	okapi	4	35:01:30	indri	6	57:56:54	okapi	4	30:45:04	potto	6	137:08:38	indri	6	46:11:03
okapi	4	77:03:22	indri	6	75:05:53	llama	4	42:59:39	okapi	4	242:22:11	potto	6	92:18:20	okapi	4	34:46:21	indri	6	54:36:42	okapi	4	30:46:52	potto	6	137:34:15	indri	6	46:05:43
okapi	4	78:43:13	indri	6	76:18:53	llama	4	43:06:09	okapi	4	244:26:02	potto	6	92:33:00	okapi	4	34:54:41	indri	6	55:11:29	okapi	4	30:49:05	potto	6	131:23:49	indri	6	46:23:53
okapi	4	77:31:35	indri	6	75:29:36	llama	4	43:21:24	okapi	4	239:25:02	potto	6	91:13:19	okapi	4	34:32:16	indri	6	54:43:59	okapi	4	30:36:35	potto	6	146:24:01	indri	6	45:49:15
okapi	4	77:26:30	indri	6	75:53:31	llama	4	43:03:30	okapi	4	240:09:53	potto	6	92:36:04	okapi	4	34:51:55	indri	6	55:13:23	okapi	4	30:47:48	potto	6	171:56:33	indri	6	45:50:45
okapi	4	78:11:11	indri	6	96:46:05	llama	4	43:16:46	okapi	4	240:53:03	potto	6	92:28:23	okapi	4	35:14:01	indri	6	55:20:27	okapi	4	30:49:11	potto	6	129:24:59	indri	6	40:43:59
okapi	4	77:19:10	indri	6	84:11:41	llama	4	43:14:58	okapi	4	240:07:06	potto	6	96:07:52	okapi	4	35:12:48	indri	6	54:12:12	okapi	4	30:36:06	potto	6	128:47:20	indri	6	46:30:21
okapi	4	78:03:52	indri	6	80:25:30	llama	4	43:07:55	okapi	4	240:39:01	potto	6	92:41:52	okapi	4	35:05:34	indri	6	55:16:32	okapi	4	30:43:56	potto	6	127:10:29	indri	6	45:54:05
okapi	4	77:25:40	indri	6	87:47:55	camel	6	41:10:03	okapi	4	239:30:33	potto	6	86:29:17	okapi	4	34:44:27	indri	6	54:42:14	okapi	4	30:33:14	potto	6	127:25:19	indri	6	46:22:14

Noncoding

2L1 (4133)			2La (6732)			2L2 (2330)			2R (17027)			2L1+2 (6463)			3L1 (2496)			3La (6208)			3L2 (1823)			3R (14323)			3L1+2 (4319)		
Machine	Cores	Time	Machine	Cores	Time	Machine	Cores	Time	Machine	Cores	Time	Machine	Cores	Time	Machine	Cores	Time	Machine	Cores	Time	Machine	Cores	Time	Machine	Cores	Time	Machine	Cores	Time
cluster	4	125:31:11	cluster	4	203:46:24	cluster	4	217:21:21	okapi	4	581:42:17	llama	6	227:23:12	cluster	4	87:55:48	camel	6	173:04:11	cluster	4	70:11:35	llama	6	361:54:46	potto	4	279:47:07
cluster	4	118:45:21	cluster	4	216:24:23	cluster	4	110:51:48	potto	6	471:45:02	llama	6	199:33:05	cluster	4	75:07:04	camel	6	171:13:15	cluster	4	70:32:17	llama	6	355:39:02	potto	4	126:33:14
cluster	4	138:01:50	cluster	4	278:58:09	cluster	4	93:35:35	potto	6	575:59:10	llama	6	203:56:24	cluster	4	83:11:04	potto	6	178:52:13	cluster	4	92:36:08	llama	6	356:04:45	potto	4	287:31:23
cluster	4	123:14:51	cluster	4	212:08:16	cluster	4	91:29:10	okapi	4	582:34:07	llama	6	194:59:37	cluster	4	86:28:45	potto	6	177:29:05	cluster	4	64:17:21	llama	6	355:10:58	potto	4	152:51:24
cluster	4	118:25:00	cluster	4	258:30:01	cluster	4	94:57:42	okapi	4	575:33:17	llama	6	217:38:11	cluster	4	72:49:50	potto	6	180:30:35	cluster	4	103:50:49	llama	6	353:35:49	potto	4	160:33:58
cluster	4	117:54:36	cluster	4	271:04:43	cluster	4	93:20:34	okapi	4	580:21:52	llama	6	200:40:22	cluster	4	86:54:38	potto	6	187:38:31	cluster	4	57:05:51	llama	6	354:30:29	potto	4	160:28:02
cluster	4	118:31:44	cluster	4	252:50:39	cluster	4	76:13:26	okapi	4	581:03:08	llama	6	194:32:47	cluster	4	91:40:41	potto	6	177:06:20	cluster	4	56:34:32	llama	6	355:06:15	indri	9	99:47:00
cluster	4	118:47:36	cluster	4	279:42:48	cluster	4	92:33:25	okapi	4	577:10:43	llama	6	203:36:59	cluster	4	92:04:16	potto	6	181:49:02	cluster	4	57:21:59	llama	6	359:58:01	indri	9	93:49:40
cluster	4	120:11:22	cluster	4	253:51:47	cluster	4	90:54:55	okapi	4	583:07:48	indri	9	239:49:30	cluster	4	97:35:20	potto	6	198:17:21	cluster	4	57:16:32	indri	9	331:12:26	indri	9	99:27:35
cluster	4	118:21:56	cluster	4	223:49:46	potto	6	75:06:28	okapi	4	580:39:45	indri	9	207:36:16	cluster	4	82:58:08	potto	8	204:56:50	cluster	4	56:40:49	indri	9	327:10:40	indri	9	98:50:56

Machine	CPU
potto	Intel Xeon Gold 6154 COU @ 3.00GHz
indri	Intel Xeon Gold 6154 COU @ 3.00GHz
okapi	Intel Xeon Gold 6230 CPU @ 2.10GHz
llama	Intel Xeon Gold 6136 CPU @ 3.00GHz
camel	Intel Xeon Gold 5118 CPU @ 2.30GHz
cluster	heterogeneous CPUs

References

1. I Gronau, MJ Hubisz, B Gulko, CG Danko, A Siepel, Bayesian inference of ancient human demography from individual genome sequences. *Nat. Genet*. **43**, 1031–1034 (2011).
2. BP Carlin, S Chib, Bayesian model choice through Markov chain Monte Carlo. *J. R. Stat. Soc. B* **57**, 473–483 (1995).
3. PJ Green, Reversible jump Markov chain Monte Carlo computation and Bayesian model determination. *Biometrika* **82**, 711–732 (1995).
4. SJ Godsill, On the relationship between Markov chain Monte Carlo methods for model uncertainty. *J. Comp. Graph. Stats.* **10**, 230–248 (2001).
5. PJ Green, T O'Hagan, Model choice with MCMC on product spaces without using pseudo-priors. *Tech. Report, Univ. Nottm*. (1998).
6. B Rannala, Z Yang, Bayes estimation of species divergence times and ancestral population sizes using DNA sequences from multiple loci. *Genetics* **164**, 1645–1656 (2003).
7. T Flouri, et al., Bayesian phylogenetic inference using relaxed-clocks and the multispecies coalescent. *Mol. Biol. Evol.* **39**, msac161 (2022).
8. Z Yang, A likelihood ratio test of speciation with gene flow using genomic sequence data. *Genom. Biol. Evol.* **2**, 200–211 (2010).
9. T Flouri, X Jiao, B Rannala, Z Yang, Species tree inference with BPP using genomic sequences and the multispecies coalescent. *Mol. Biol. Evol.* **35**, 2585–2593 (2018).
10. T Flouri, X Jiao, B Rannala, Z Yang, A Bayesian implementation of the multispecies coalescent model with introgression for phylogenomic analysis. *Mol. Biol. Evol.* **37**, 1211–1223 (2020).
11. TH Jukes, CR Cantor, *Evolution of protein molecules*. (Academic Press, New York), pp. 21–123 (1969).
12. R Burgess, Z Yang, Estimation of hominoid ancestral population sizes under Bayesian coalescent models incorporating mutation rate variation and sequencing errors. *Mol. Biol. Evol.* **25**, 1979–1994 (2008).
13. M Hasegawa, H Kishino, T Yano, Dating the human-ape splitting by a molecular clock of mitochondrial DNA. *J. Mol. Evol.* **22**, 160–174 (1985).
14. P Beerli, Comparison of Bayesian and maximum-likelihood inference of population genetic parameters. *Bioinformatics* **22**, 341–345 (2006).
15. MC Fontaine, et al., Extensive introgression in a malaria vector species complex revealed by phylogenomics. *Science* **347**, 1258524 (2015).
16. Y Thawornwattana, D Dalquen, Z Yang, Coalescent analysis of phylogenomic data confidently resolves the species relationships in the *Anopheles gambiae* species complex. *Mol. Biol. Evol.* **35**, 2512–2527 (2018).
17. J Huang, Y Thawornwattana, T Flour, J Mallet, Z Yang, Inference of gene flow between species under misspecified models. *Mol. Biol. Evol.* **39**, msac237 (2022).
